# Cooking oil/fat consumption and deaths from cardiometabolic diseases and other causes: prospective analysis of 521,120 individuals

**DOI:** 10.1186/s12916-021-01961-2

**Published:** 2021-04-15

**Authors:** Yu Zhang, Pan Zhuang, Fei Wu, Wei He, Lei Mao, Wei Jia, Yiju Zhang, Xiaoqian Chen, Jingjing Jiao

**Affiliations:** 1 Department of Food Science and Nutrition, Zhejiang Key Laboratory for Agro-Food Processing, Fuli Institute of Food Science, College of Biosystems Engineering and Food Science, Zhejiang University, Hangzhou, 310058 Zhejiang China; 2 Department of Nutrition, School of Public Health, and Department of Nutrition of Affiliated Second Hospital, Zhejiang University School of Medicine, 866 Yuhangtang Road, Hangzhou, 310058 Zhejiang China; 3 Department of Medical Epidemiology and Biostatistics, Karolinska Institutet, Huddinge, SE-171 77 Stockholm, Sweden

**Keywords:** Cooking oils, Cardiometabolic mortality, Total mortality, AARP Diet and Health Study

## Abstract

**Background:**

Increasing evidence highlights healthy dietary patterns and links daily cooking oil intake with chronic diseases including cardiovascular disease (CVD) and diabetes. However, food-based evidence supporting the consumption of cooking oils in relation to total and cardiometabolic mortality remains largely absent. We aim to prospectively evaluate the relations of cooking oils with death from cardiometabolic (CVD and diabetes) and other causes.

**Methods:**

We identified and prospectively followed 521,120 participants aged 50–71 years from the National Institutes of Health-American Association of Retired Persons Diet and Health Study. Individual cooking oil/fat consumption was assessed by a validated food frequency questionnaire. Hazard ratios (HRs) and 95% confidence intervals (CIs) were estimated for mortality through the end of 2011.

**Results:**

Overall, 129,328 deaths were documented during a median follow-up of 16 years. Intakes of butter and margarine were associated with higher total mortality while intakes of canola oil and olive oil were related to lower total mortality. After multivariate adjustment for major risk factors, the HRs of cardiometabolic mortality for each 1-tablespoon/day increment were 1.08 (95% CI 1.05–1.10) for butter, 1.06 (1.05–1.08) for margarine, 0.99 (0.95–1.03) for corn oil, 0.98 (0.94–1.02) for canola oil, and 0.96 (0.92–0.99) for olive oil. Besides, butter consumption was positively associated with cancer mortality. Substituting corn oil, canola oil, or olive oil for equal amounts of butter and margarine was related to lower all-cause mortality and mortality from certain causes, including CVD, diabetes, cancer, respiratory disease, and Alzheimer’s disease.

**Conclusions:**

Consumption of butter and margarine was associated with higher total and cardiometabolic mortality. Replacing butter and margarine with canola oil, corn oil, or olive oil was related to lower total and cardiometabolic mortality. Our findings support shifting the intake from solid fats to non-hydrogenated vegetable oils for cardiometabolic health and longevity.

## Background

Cooking oils/fats are known as edible oils from vegetable or animal origin and used for cuisine or salad preparation worldwide. To meet consumption needs, global vegetable oil production was closed to 198 million metric tons in 2018–2019. For animal or artificial cooking fats, 187.1 million Americans used margarine or margarine spread while butter consumption in the USA reached 5.8 pounds per capita in 2019 [1]. Growing controversy focused on the role of cooking oils/fats in the incidence of various chronic disorders including cardiovascular disease (CVD) events. Importantly, the well-known functions of saturated fatty acids (SFAs), monounsaturated fatty acids (MUFAs), or polyunsaturated fatty acids (PUFAs) could not apparently translate to cardiometabolic health effects of cooking oils/fats [2]. Thus, increasing evidence supports shifting away from isolated fatty acids toward food-based patterns for linking dietary cooking oils/fats with all-cause mortality, fatal and nonfatal CVD events [3, 4]. Vegetable cooking oils are regarded as the healthier choice as they contain more unsaturated fatty acids than animal oils. Canola oil and corn oil may ameliorate blood lipid profile and protect against CVD risk factors [5, 6], whereas butter raises total and LDL cholesterol levels [7]. Canola oil- or olive oil-enriched diet could improve glycemic control in patients with type 2 diabetes [6, 8]. However, only a few studies have provided weak evidence of cooking oil/fat consumption in relation to all-cause and cardiometabolic mortality [9–11]. The associations of lard, canola oil, and corn oil consumption with cardiometabolic mortality remain lacking.

To fill these gaps, we assessed the long-term associations of 6 typical cooking oils/fats, including butter, margarine, lard, corn oil, canola oil, and olive oil, with all-cause, cardiometabolic, and other major cause-specific mortality in the National Institutes of Health-American Association of Retired Persons (NIH-AARP) Diet and Health Study.

## Methods

### Study population

The NIH-AARP Diet and Health Study is a large prospective cohort consisting of 617,119 US men and women aged 50–71 years. At baseline in 1995–1996, validated questionnaires were mailed to 3.5 million AARP members from 6 US states (California, Florida, Louisiana, New Jersey, North Carolina, and Pennsylvania) and 2 metropolitan areas (Atlanta, Georgia, and Detroit, Michigan) to collect data on demographics, lifestyle, and dietary characteristics. All participants provided written informed consent. Among 567,169 participants who completed the questionnaires, we excluded participants who were proxy responders, had duplicate records, decided to withdraw, moved or died before entry, and had null person-years of follow-up or extreme total energy intake (< 800 or > 4200 kcal/day for men and < 600 or > 3500 kcal/day for women [12]). Finally, 521,120 persons were selected (Additional file 1: Fig. S1), which was approved by the Institutional Review Board of the National Cancer Institute.

### Assessment of diet and cooking oils/fats

Dietary intake was assessed at baseline using a 124-item self-administered food frequency questionnaire (FFQ) developed as the diet history questionnaire (DHQ) and validated by National Cancer Institute [13]. The frequency and portion sizes of food consumption were recorded during the past year. Some questions were asked to collect the frequency of oils/fats used in cooking and added after cooking, such as “How often was oil, butter, or margarine used to fry or saute the vegetables, eggs, or meat you ate?” followed by options from “never” to “4 or more times per day” and “When you ate each of the foods listed in this table, how often was butter or margarine added after cooking or at table?” followed by options from “almost never or never” to “more than half the time.” Participants were also asked to select the types of oils/fats they regularly used, including butter, margarine, lard, corn oil, canola oil, and olive oil. Portion sizes for individual oils/fats were estimated based on the 1994–1996 USDA Continuing Survey of Food Intakes by Individuals (CSFII) [14] and intakes of cooking oils/fats were then calculated by multiplying the frequency of consumption with the corresponding portion size derived from CSFII. Cooking oils/fats included in foods were also accounted for by asking questions such as the frequency of consuming butter or margarine on bread or rolls. Three typical solid fats (butter, margarine, and lard) and 3 commonly consumed vegetable oils (olive oil, canola oil, and corn oil) were calculated and analyzed in our study. The Healthy Eating Index (HEI)-2015 score was established to assess the adherence to an overall healthy dietary pattern according to US Dietary Guideline 2015–2020. The HEI-2015 is composed of 13 components, including total fruit, whole fruit, total vegetables, greens and beans, whole grains, dairy, total protein foods, seafood and plant proteins, fatty acids, refined grains, sodium, added sugars, and saturated fats [15]. Scores are assigned to each component by comparing the density (the amount of dietary component per 1000 kcal) to the relevant standards [15].

### Mortality

All the participants were followed for address changes via the US Postal Service National Change of Address database, responses to study-related mailings, and direct notifications from cohort members. Deaths were identified by annual linkage to the Social Security Administration Death Master File and confirmed by follow-up searches of the National Death Index Plus. The *International Classification of Diseases 9th and 10th Revision* codes were used to classify death causes (Additional file 1: Table S1). Follow-up was calculated from the return date of the baseline questionnaire to the time of death or the end of follow-up (31 December 2011), whichever occurred earlier. The complete follow-up rate for mortality exceeds 99% in this cohort study.

### Statistical analysis

Intakes of individual cooking oils/fats were expressed as the functions of energy density (g 2000 kcal^−1^ day^−1^) using the nutrient density method [16]. We used Cox proportional hazards regression models to estimate hazard ratios (HRs) and 95% confidence intervals (CIs) for mortality. Model 1 was adjusted for age and sex at baseline. Model 2 was further adjusted for race, marital status, education, household income, body mass index (BMI), alcohol, smoking, vigorous physical activity, usual activity at work, perceived health condition, and history of cancer, heart disease, stroke, and diabetes. Our final multivariate model 3 was additionally adjusted for HEI-2015 (with no fat components), total energy intake, and consumption of remaining oils where appropriate. Tests for linear trend were performed by assigning median values to corresponding categories of intake and modeling the values as continuous variables. We also estimated the associations of hypothetical substitution of 1 tablespoon/day olive oil, canola oil, or corn oil for the equivalent amounts of butter and margarine with mortality by simultaneously including individual cooking oils as continuous variables and total cooking oil intake in the same multivariable model (substitution model), which also contained total energy intake, HEI, and other non-dietary covariates. Total oil/fat intake was held constant in this model. By leaving butter or margarine out of this model, regression coefficients of the remaining oils bear the interpretations as the theoretical effects of substituting one of these oils for the equivalent amounts of butter or margarine while holding other oils unchanged. A fixed 1-tablespoon/day increase corresponds to an increment of approximately 14 g/day butter/margarine or 8 g/day vegetable oils [9, 17].

We further separately analyzed the associations for stick margarine and tub/soft margarine, respectively. Subgroup analyses were also conducted according to important potential effect modifiers and *P* values for interactions were tested by the likelihood-ratio test. Sensitivity analyses were performed by excluding participants who had extreme BMI (< 18.5 or > 40 kg/m^2^); using the propensity-score adjustment [18] to further control for potential residual confounding from measured variables; further adjusting for history of hypertension and hypercholesteremia and the use of aspirin and multivitamins; excluding the initial 4 years of follow-up; excluding those who had CVD, cancer, or diabetes at baseline; or ending up the follow-up at the year 2004 (midpoint, 8 years of follow-up). We also tested whether the associations were affected by the use of cholesterol-lowering medications among persons who provided this information in the resurvey (*n* = 293,918).

Statistical analyses were performed with SAS version 9.4 (SAS Institute, Cary, NC, USA). Tests were two-sided and the significance was defined as *P* < 0.05.

## Results

### Population characteristics and cooking oil/fat consumption

During an average of 16 years of follow-up (7,307,097 person-years), 129,328 individuals died, including 85,037 men and 44,291 women. At baseline, participants with higher butter consumption were less likely to be married, have prevalent heart disease, and use aspirin, and they had lower protein intake and lower HEI scores. Participants with higher margarine consumption were more likely to have a higher BMI, use aspirin, and have heart disease, stroke, and diabetes, and they had lower household income and lower alcohol intake. The median intakes in the highest tertile among consumers were 13.7 g 2000 kcal^− 1^ day^−1^ for butter and 20.6 g 2000 kcal^−1^ day^−1^ for margarine, respectively (Table 1). Participant characteristics according to corn oil, canola oil, and olive oil consumption are shown in Additional file 1: Table S2. The Spearman correlations between individual cooking oil/fat consumption are presented in Additional file 1: Table S3.

**Table 1:** Baseline characteristics of participants from the NIH-AARP Diet and Health Study according to butter and margarine consumption

	Butter consumption				Margarine consumption			
Characteristics	Non-consumers	T1	T2	T3	Non-consumers	T1	T2	T3
Range (g 2000 kcal^−1^ d^−1^)	0	≤ 3.1	3.2–8.5	≥ 8.6	0	≤5.7	5.8–13.7	≥13.8
*n*	303,987	72,377	72,378	72,378	134,374	128,915	128,916	128,915
Age (y)	63.0	62.2	62.5	63.1	62.5	62.3	62.8	63.6
Male (%)	58.4	65.1	61.2	51.7	56.3	64.3	62.7	52.0
BMI (kg/m^2^)	26.4	26.1	26.5	26.4	26.1	26.2	26.6	26.5
Race (%)								
White	92.3	89.4	91.3	92.6	91.7	89.9	92.5	93.0
Black	3.8	3.1	3.6	3.4	3.6	3.4	3.5	4.0
Hispanic	1.6	3.0	2.1	1.3	1.7	2.7	1.7	1.1
Asian	0.9	2.6	1.4	0.9	1.2	2.1	0.8	0.5
Married (%)	69.9	68.1	67.9	63.0	65.5	69.7	71.0	67.6
Annual household income (USD)^a^	47,243	51,690	50,330	49,925	50,282	50,557	48,047	46,077
College graduate or postgraduate (%)	37.3	47.5	39.8	36.8	40.1	44.6	38.2	33.2
Current smoker (%)	10.8	9.1	13.2	16.1	12.9	9.6	12.0	12.1
Physical activity, ≥ 5 times/wk (%)	19.4	22.1	17.6	16.3	19.8	21.6	18.0	17.1
Heart disease (%)	15.7	12.9	11.5	10.5	12.2	13.8	14.3	15.7
Stroke (%)	2.2	1.8	1.9	2.1	1.9	2.0	2.1	2.5
Cancer (%)	9.1	8.4	8.6	9.7	9.0	8.4	8.9	9.7
Diabetes (%)	9.6	7.4	8.3	9.2	7.8	7.7	9.3	11.6
Fair or poor health (%)	13.5	10.7	12.0	13.5	12.0	11.2	12.9	15.4
Currently using multivitamins (%)	55.8	56.6	55.2	54.8	56.1	56.4	55.0	55.2
Daily use of aspirin (%)	16.2	14.4	13.0	11.9	13.5	15.2	15.2	15.8
Daily dietary intake								
Total energy (kcal/d)	1666.6	1651.6	1759.3	1749.3	1711.1	1649.3	1733.4	1661.4
Alcohol from alcoholic drinks (g/d)	1.5	2.5	2.7	2.0	2.2	2.1	2.0	1.2
Total protein (% of energy)	15.5	15.6	15.2	14.6	15.0	15.7	15.5	15.2
Total fat (% of energy)	29.8	26.6	31.1	35.0	31.1	26.6	30.4	32.8
Butter	0.0	1.0	5.5	13.7	4.0	0.3	0.0	0.0
Margarine	10.4	1.1	4.3	0.0	0.0	2.4	9.3	20.6
Corn oil	0.0	0.0	0.0	0.0	0.0	0.0	0.0	0.0
Canola oil	0.0	0.0	0.0	0.0	0.0	0.0	0.0	0.0
Olive oil	0.0	0.0	0.0	0.0	0.0	0.0	0.0	0.0
Healthy Eating Index score	70.5	70.4	66.4	61.6	65.8	70.1	68.6	70.2

Data are medians or percentages. *BMI* body mass index, *T* tertile

^a^Household income in 1999

### All-cause mortality

**Fig. 1:**
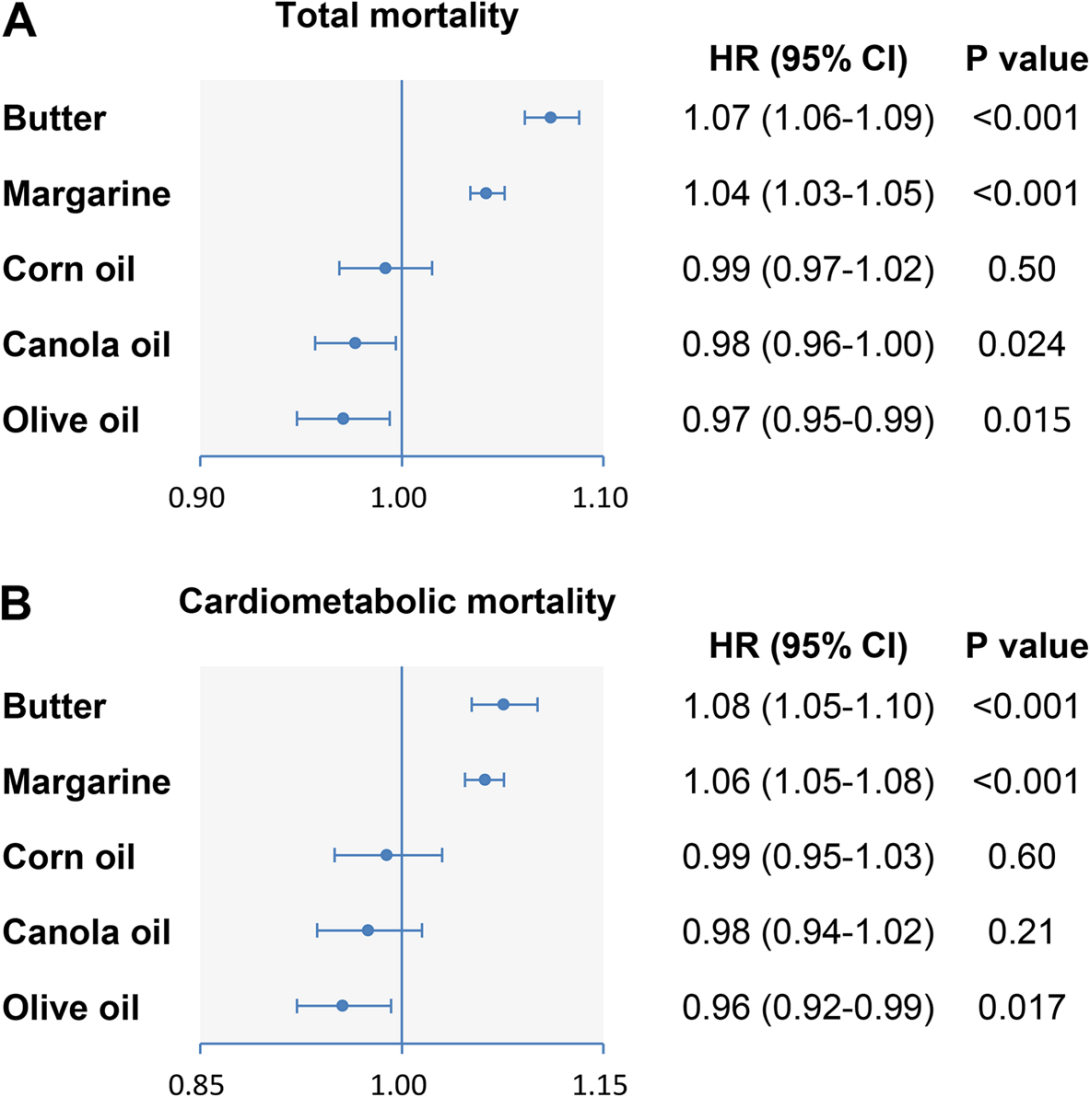
Multivariable-adjusted hazard ratios of total and cardiometabolic mortality for 1-tablespoon/day increment in cooking oil/fat consumption. Forest plots show the multivariable HRs of total (**a**) and cardiometabolic (**b**) mortality associated with 1-tablespoon/day increment in butter, margarine, corn oil, canola oil, and olive oil consumption. HRs were adjusted for age, sex, BMI, race, education, marital status, household income, smoking, alcohol, vigorous physical activity, usual activity at work, perceived health condition, history of heart disease, stroke, diabetes, and cancer at baseline, Healthy Eating Index-2015, total energy intake, and consumption of remaining oils where appropriate (butter, margarine, lard, corn oil, canola oil, olive oil, and other vegetable oils). Horizontal lines represent 95% CIs

Butter and margarine consumption was strongly associated with higher all-cause mortality in all multivariable-adjusted models. In contrast, intakes of canola oil and olive oil were both inversely associated with all-cause mortality. Corn oil consumption was related to higher all-cause mortality after adjusting for age and sex, but the association became non-significant after adjusting further covariates (Table 2). Compared to non-consumers, the multivariable HRs of all-cause mortality in the highest categories were 1.09 (95% CI, 1.07–1.11) for butter (*P*-trend < 0.001), 1.07 (95% CI, 1.05–1.09) for margarine (*P*-trend < 0.001), 0.97 (95% CI, 0.95–0.99) for canola oil (*P*-trend < 0.001), and 0.96 (95% CI, 0.95–0.98) for olive oil (*P*-trend < 0.001) (Table 2). Every 1-tablespoon/day increment of butter or margarine consumption was related to 7% and 4% higher all-cause mortality, respectively. In contrast, each 1-tablespoon/day increment of canola oil or olive oil consumption was associated with 2% and 3% of reductions in all-cause mortality, respectively (Fig. 1a).

**Table 2:** HRs (95% CIs) of all-cause mortality according to cooking oil/fat consumption

	Categories of individual cooking oil/fat consumption				
	Non-consumers	T1	T2	T3	***P*** trend
**Butter**					
Median intake (IQR)	0	1.0 (0.4–2.0)	5.5 (4.2–6.9)	13.7 (10.7–18.8)	
Death cases/*n*	75,826/303,987	15,792/72,377	17,915/72,378	19,795/72,378	
Person-years	4,262,749	1,030,708	1,014,202	999,439	
Model 1^a^	1.00	0.88 (0.86–0.89)	1.02 (1.00–1.03)	1.14 (1.13–1.16)	< 0.001
Model 2^b^	1.00	0.96 (0.94–0.97)	1.04 (1.02–1.06)	1.12 (1.10–1.14)	< 0.001
Model 3^c^	1.00	0.98 (0.96–1.00)	1.05 (1.03–1.06)	1.09 (1.07–1.11)	< 0.001
**Margarine**					
Median intake (IQR)	0	2.4 (0.9–4.0)	9.3 (7.4–11.4)	20.6 (16.7–26.6)	
Death cases/*n*	32,633/134,374	29,070/128,915	32,583/128,916	35,042/128,915	
Person-years	1,888,086	1,829,523	1,804,411	1,785,077	
Model 1^a^	1.00	0.90 (0.89–0.92)	1.01 (0.99–1.02)	1.08 (1.06–1.09)	< 0.001
Model 2^b^	1.00	0.94 (0.92–0.95)	0.99 (0.97–1.00)	1.00 (0.99–1.02)	< 0.001
Model 3^c^	1.00	0.99 (0.97–1.01)	1.03 (1.01–1.05)	1.07 (1.05–1.09)	< 0.001
**Corn oil**					
Median intake (IQR)	0	0.4 (0.2–0.5)	1.1 (0.8–1.4)	3.4 (2.4–5.5)	
Death cases/*n*	98,499/399,360	9400/40586	10,240/40,587	11,189/40,587	
Person-years	5,605,569	574,055	567,538	559,935	
Model 1^a^	1.00	0.95 (0.93–0.97)	1.03 (1.01–1.05)	1.12 (1.10–1.14)	< 0.001
Model 2^b^	1.00	0.96 (0.94–0.98)	0.99 (0.97–1.01)	1.02 (1.00–1.04)	0.21
Model 3^c^	1.00	0.97 (0.94–0.99)	0.98 (0.96–1.00)	0.99 (0.97–1.01)	0.092
**Canola oil**					
Median intake (IQR)	0	0.4 (0.2–0.5)	1.0 (0.8–1.3)	3.2 (2.3–5.3)	
Death cases/*n*	95,507/376,913	10,571/48,069	11,129/48,069	12,121/48,069	
Person-years	5,269,352	684,537	680,475	672,733	
Model 1^a^	1.00	0.88 (0.86–0.89)	0.91 (0.89–0.92)	0.98 (0.96–0.99)	< 0.001
Model 2^b^	1.00	0.94 (0.93–0.96)	0.96 (0.94–0.97)	0.97 (0.95–0.99)	< 0.001
Model 3^c^	1.00	0.98 (0.95–1.00)	0.97 (0.95–0.99)	0.97 (0.95–0.99)	< 0.001
**Olive oil**					
Median intake (IQR)	0	0.4 (0.3–0.5)	1.2 (0.9–1.5)	3.8 (2.6–6.2)	
Death cases/*n*	91,948/353,766	11,878/55,784	12,386/55,785	13,116/55,785	
Person-years	4,930,793	796,402	793,033	786,869	
Model 1^a^	1.00	0.85 (0.84–0.87)	0.87 (0.86–0.89)	0.91 (0.89–0.93)	< 0.001
Model 2^b^	1.00	0.94 (0.92–0.96)	0.96 (0.94–0.98)	0.97 (0.95–0.99)	< 0.001
Model 3^c^	1.00	0.96 (0.94–0.99)	0.97 (0.95–0.98)	0.96 (0.95–0.98)	< 0.001

HRs (95% CIs) were estimated using Cox proportional hazards models. *CI* confidence interval, *HR* hazard ratio, *T* tertile

^a^Adjusted for age and sex

^b^Additionally adjusted for BMI (in kg/m^2^; < 18.5, 18.5 to 25, 25 to 30, 30 to 35, ≥ 35, or missing), race (white, black, Hispanic/Asian/Pacific Islander/American Indian/Alaskan native, or unknown/missing), education (less than high school, high school graduate, some college, college graduate, or unknown/missing), marital status (married/living as married or widowed/divorced/separated/never married/unknown), household income (quintiles), smoking (never smoked; quit, ≤ 20 cigarettes a day; quit, > 20 cigarettes a day; currently smoking, ≤ 20 cigarettes a day; currently smoking, > 20 cigarettes a day; or unknown), alcohol (0, 0.1–4.9, 5.0–29.9, or ≥ 30 g/day), vigorous physical activity (never/rarely, 1–3 times/month, 1–2 times/week, 3–4 times/week, ≥ 5 times/week, or unknown/missing), usual activity at work (sit all day, sit much of the day/walk sometimes, stand/walk often/no lifting, lift/carry light loads, and carry heavy loads), perceived health condition (excellent, very good, good, fair or poor),and history of heart disease (yes or no), stroke (yes or no), diabetes (yes or no), and cancer (yes or no) at baseline

^c^Additionally adjusted for Healthy Eating Index-2015, total energy intake, and consumption of remaining oils where appropriate (butter, margarine, lard, corn oil, canola oil, olive oil, and other vegetable oils)

### Cardiometabolic mortality

**Fig. 2:**
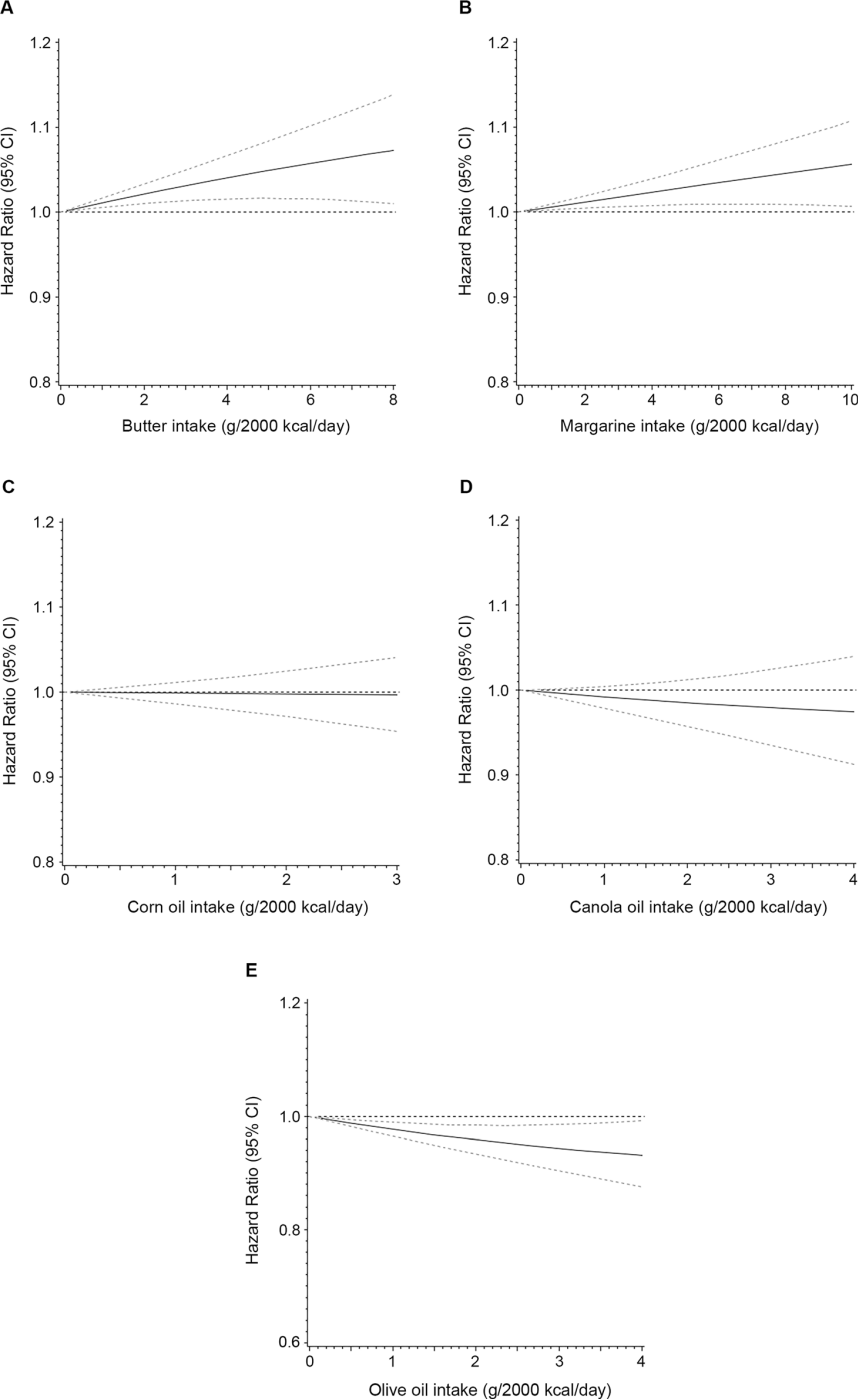
Cubic spline curves for the association between individual cooking oil intakes and cardiometabolic mortality. Hazard ratios are based on Cox proportional hazards models adjusted for age, sex, BMI, race, education, marital status, household income, smoking, alcohol, vigorous physical activity, usual activity at work, perceived health condition, history of heart disease, stroke, diabetes, and cancer at baseline, Healthy Eating Index-2015, total energy intake, and consumption of remaining oils where appropriate (butter, margarine, lard, corn oil, canola oil, olive oil, and other vegetable oils)

The consumption of butter and margarine was positively associated with CVD mortality after multivariate adjustment, whereas olive oil intake was inversely associated with CVD mortality (Table 3). Compared with non-consumers, participants in the highest tertile of olive oil consumption had 5% (HR = 0.95, 95% CI 0.92–0.99; *P*-trend = 0.001) lower CVD mortality, but those in the highest tertiles of butter and margarine consumption had 8% (HR = 1.08, 95% CI 1.05–1.12; *P*-trend< 0.001) and 10% (HR = 1.10, 95% CI 1.06–1.14; *P*-trend< 0.001) higher CVD mortality, respectively. Canola oil consumption was marginally associated with lower CVD mortality (*P*-trend = 0.052), while corn oil intake was not related to CVD mortality. Similar associations were also observed for heart disease mortality (Additional file 1: Table S4). Besides, borderline trends toward lower and higher stroke mortality were observed for corn oil (*P*-trend = 0.061) and butter (*P*-trend = 0.059) consumption, respectively. For diabetes mortality, we detected positive associations for butter and margarine consumption. Compared with non-consumers, participants in the highest tertiles of butter and margarine consumption had 18% (HR = 1.18, 95% CI 1.06–1.32; *P*-trend = 0.0041) and 12% (HR = 1.12, 95% CI 1.00–1.26; *P*-trend = 0.047) higher diabetes mortality, respectively. In contrast, olive oil consumption was inversely related to diabetes mortality (HR comparing the highest tertile with non-consumers = 0.87, 95% CI 0.77–0.99; *P*-trend = 0.019). Overall, each 1-tablespoon/day increment of butter or margarine consumption was associated with 8% and 6% higher cardiometabolic mortality, respectively, while every 1-tablespoon/day increment of olive oil consumption was related to 4% decreased cardiometabolic mortality (Fig. 1b). Restricted-cubic-spline regression yielded similar results (Fig. 2a–e).

**Table 3:** HRs (95% CIs) of CVD and diabetes mortality according to cooking oil/fat consumption

	Categories of individual cooking oil/fat consumption				
	Non-consumers	T1	T2	T3	***P*** trend
**Cardiovascular disease mortality**					
**Butter**					
Death cases/*n*	23,406/303,987	4623/72,377	5213/72,378	5505/72,378	
Model 1^a^	1.00	0.83 (0.80–0.86)	0.96 (0.93–0.99)	1.04 (1.01–1.07)	< 0.001
Model 2^b^	1.00	0.93 (0.90–0.96)	1.02 (0.99–1.05)	1.07 (1.04–1.11)	< 0.001
Model 3^c^	1.00	0.96 (0.93–1.00)	1.04 (1.01–1.08)	1.08 (1.05–1.12)	< 0.001
**Margarine**					
Death cases/*n*	9305/134,374	8630/128,915	9901/128,916	10,911/128,915	
Model 1^a^	1.00	0.93 (0.90–0.96)	1.06 (1.03–1.09)	1.17 (1.14–1.21)	< 0.001
Model 2^b^	1.00	0.95 (0.92–0.98)	1.02 (0.99–1.05)	1.04 (1.01–1.07)	< 0.001
Model 3^c^	1.00	1.01 (0.97–1.04)	1.06 (1.02–1.09)	1.10 (1.06–1.14)	< 0.001
**Corn oil**					
Death cases/*n*	29,443/399,360	2830/40,586	3068/40,587	3406/40,587	
Model 1^a^	1.00	0.97 (0.93–1.01)	1.03 (0.99–1.07)	1.14 (1.10–1.18)	< 0.001
Model 2^b^	1.00	0.99 (0.96–1.03)	1.00 (0.97–1.04)	1.02 (0.99–1.06)	0.22
Model 3^c^	1.00	1.01 (0.96–1.05)	0.99 (0.95–1.03)	1.00 (0.96–1.03)	0.78
**Canola oil**					
Death cases/*n*	28,520/376,913	3149/48,069	3362/48,069	3716/48,069	
Model 1^a^	1.00	0.89 (0.85–0.92)	0.93 (0.89–0.96)	1.01 (0.97–1.04)	0.70
Model 2^b^	1.00	0.96 (0.92–1.00)	0.97 (0.94–1.01)	0.97 (0.94–1.01)	0.080
Model 3^c^	1.00	0.99 (0.95–1.04)	0.98 (0.94–1.02)	0.97 (0.94–1.00)	0.052
**Olive oil**					
Death cases/*n*	27,962/353,766	3377/55,784	3578/55,785	3830/55,785	
Model 1^a^	1.00	0.81 (0.78–0.84)	0.84 (0.81–0.87)	0.88 (0.85–0.91)	< 0.001
Model 2^b^	1.00	0.92 (0.89–0.96)	0.95 (0.92–0.98)	0.95 (0.92–0.98)	0.002
Model 3^c^	1.00	0.93 (0.89–0.97)	0.95 (0.92–0.99)	0.95 (0.92–0.99)	0.001
**Diabetes mortality**					
**Butter**					
Death cases/*n*	389/72,377	427/72,378	537/72,378	3512/72,378	
Model 1^a^	1.00	0.75 (0.67–0.83)	0.84 (0.76–0.94)	1.10 (1.00–1.21)	0.062
Model 2^b^	1.00	0.93 (0.84–1.04)	0.96 (0.86–1.06)	1.11 (1.01–1.22)	0.037
Model 3^c^	1.00	0.97 (0.86–1.09)	0.99 (0.89–1.11)	1.18 (1.06–1.32)	0.004
**Margarine**					
Death cases/*n*	782/134,374	732/128,915	896/128,916	1102/128,915	
Model 1^a^	1.00	0.94 (0.85–1.04)	1.16 (1.05–1.27)	1.44 (1.32–1.58)	< 0.001
Model 2^b^	1.00	0.97 (0.88–1.07)	1.03 (0.93–1.13)	1.06 (0.96–1.16)	0.091
Model 3^c^	1.00	1.05 (0.93–1.18)	1.08 (0.97–1.21)	1.12 (1.00–1.26)	0.047
**Corn oil**					
Death cases/*n*	2670/399,360	235/40,586	296/40,587	311/40,587	
Model 1^a^	1.00	0.88 (0.77–1.00)	1.09 (0.97–1.23)	1.15 (1.03–1.30)	0.009
Model 2^b^	1.00	0.94 (0.82–1.08)	1.06 (0.94–1.19)	0.95 (0.85–1.07)	0.56
Model 3^c^	1.00	0.96 (0.83–1.12)	1.02 (0.90–1.16)	0.95 (0.84–1.07)	0.41
**Canola oil**					
Death cases/*n*	2560/376,913	270/48,069	325/48,069	357/48,069	
Model 1^a^	1.00	0.84 (0.74–0.95)	1.00 (0.89–1.12)	1.09 (0.97–1.21)	0.14
Model 2^b^	1.00	0.99 (0.87–1.12)	1.10 (0.98–1.23)	0.99 (0.88–1.10)	0.95
Model 3^c^	1.00	1.07 (0.92–1.24)	1.08 (0.96–1.22)	0.99 (0.88–1.11)	0.96
**Olive oil**					
Death cases/*n*	2635/353,766	257/55,784	309/55,785	311/55,785	
Model 1^a^	1.00	0.64 (0.56–0.73)	0.76 (0.67–0.85)	0.75 (0.67–0.84)	< 0.001
Model 2^b^	1.00	0.85 (0.75–0.97)	0.97 (0.86–1.09)	0.87 (0.77–0.98)	0.022
Model 3^c^	1.00	0.84 (0.72–0.98)	0.94 (0.83–1.06)	0.87 (0.77–0.99)	0.019

HRs (95% CIs) were estimated using Cox proportional hazards models. *CI* confidence interval, *HR* hazard ratio, *T* tertile

^a^Adjusted for age and sex

^b^Additionally adjusted for BMI (in kg/m^2^; < 18.5, 18.5 to 25, 25 to 30, 30 to 35, ≥ 35, or missing), race (white, black, Hispanic/Asian/Pacific Islander/American Indian/Alaskan native, or unknown/missing), education (less than high school, high school graduate, some college, college graduate, or unknown/missing), marital status (married/living as married or widowed/divorced/separated/never married/unknown), household income (quintiles), smoking (never smoked; quit, ≤ 20 cigarettes a day; quit, > 20 cigarettes a day; currently smoking, ≤ 20 cigarettes a day; currently smoking, > 20 cigarettes a day; or unknown), alcohol (0, 0.1–4.9, 5.0–29.9, or ≥ 30 g/day), vigorous physical activity (never/rarely, 1–3 times/month, 1–2 times/week, 3–4 times/week, ≥5 times/week, or unknown/missing), usual activity at work (sit all day, sit much of the day/walk sometimes, stand/walk often/no lifting, lift/carry light loads, and carry heavy loads), perceived health condition (excellent, very good, good, fair or poor), and history of heart disease (yes or no), stroke (yes or no), diabetes (yes or no), and cancer (yes or no) at baseline

^c^Additionally adjusted for Healthy Eating Index-2015, total energy intake, and consumption of remaining oils where appropriate (butter, margarine, lard, corn oil, canola oil, olive oil, and other vegetable oils)

### Other non-cardiometabolic mortality

For other cause-specific mortality, butter consumption was associated with higher mortality from cancer, respiratory disease (RD), kidney disease, and chronic liver disease. Intake of margarine was related to higher RD and kidney disease mortality. Nonetheless, canola oil intake turned to be inversely associated with RD and infection mortality, while higher olive oil consumption was related to lower mortality from RD and Alzheimer’s disease (AD). We observed a borderline trend toward higher consumption of corn oil in relation to lower AD mortality (*P*-trend = 0.061) (Additional file 1: Table S5). Compared with non-consumers, lard consumers had 4% and 13% higher all-cause and RD mortality, respectively (Additional file 1: Table S6).

### Substitution for butter and margarine

**Fig. 3:**
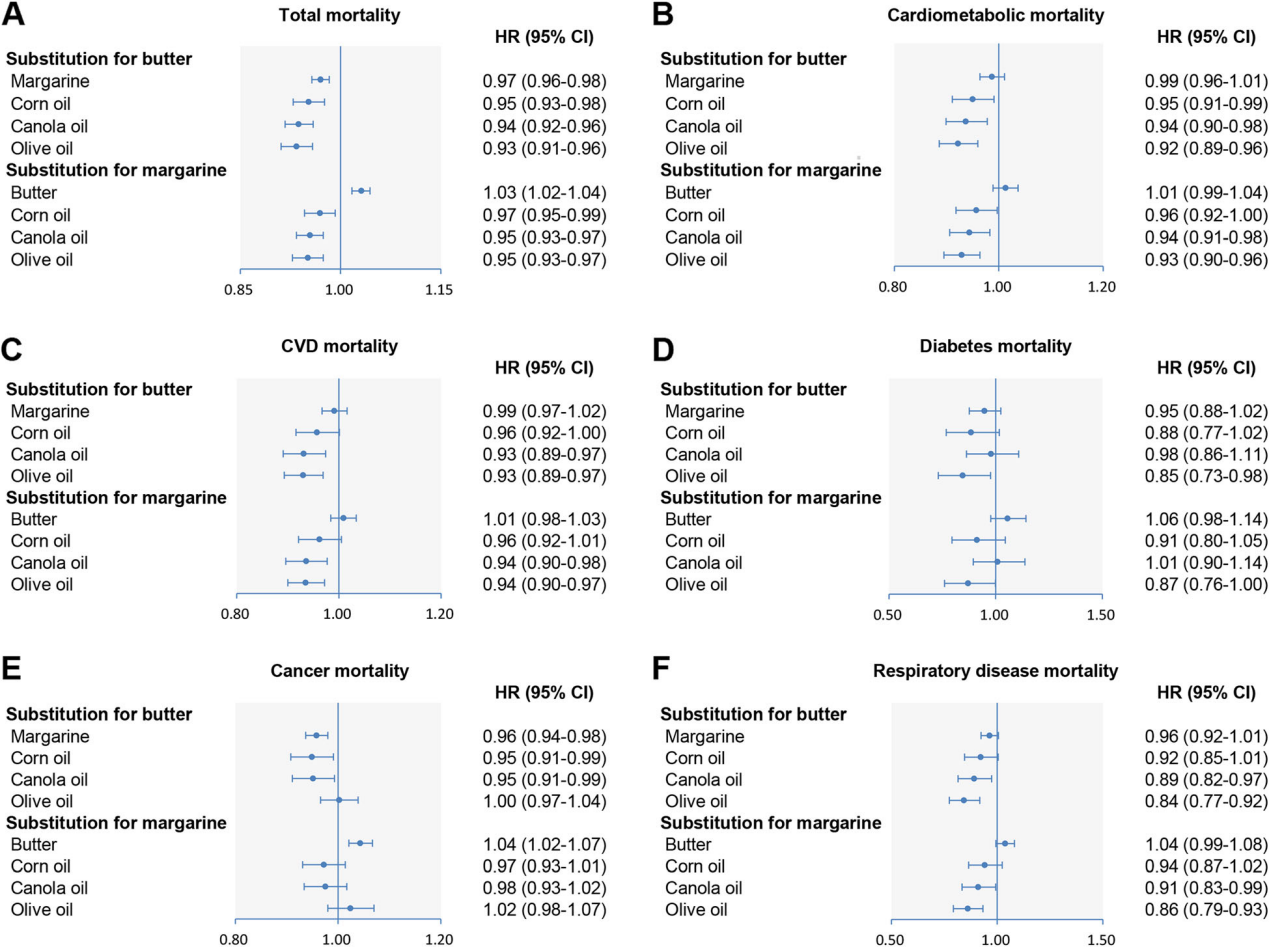
Multivariable-adjusted hazard ratios of total and cause-specific mortality by substitution of 1 tablespoon/day individual cooking oils/fats for butter and margarine. Forest plots show the multivariable HRs of total and cause-specific mortality associated with substitution of 1 tablespoon/day individual cooking oils/fats for equivalent amounts of butter and margarine. HRs were adjusted for age, sex, BMI, race, education, marital status, household income, smoking, alcohol, vigorous physical activity, usual activity at work, perceived health condition, history of heart disease, stroke, diabetes, and cancer at baseline, Healthy Eating Index-2015, and total energy intake. Horizontal lines represent 95% CIs

Substituting 1 tablespoon/day (8 g/day) corn oil, canola oil, or olive oil for equivalent amounts of butter was associated with 5%, 6%, and 7% lower all-cause mortality, respectively. Likewise, substituting 1 tablespoon/day corn oil, canola oil, or olive oil for equivalent amounts of butter was associated with 5%, 6%, and 8% lower cardiometabolic mortality, respectively. For the cause-specific mortality by the replacement of butter, each 1-tablespoon/day increment of canola oil was associated with 7%, 5%, and 11% of reductions in CVD, cancer, and RD mortality, respectively, and each 1-tablespoon/day increment of olive oil was related to 7%, 16%, 21%, and 15% lower mortality from CVD, RD, AD, and diabetes, respectively. Besides, we detected 5% lower cancer mortality when replacing 1 tablespoon/day butter with corn oil (Fig. 3 and Additional file 1: Table S7).

When replacing margarine, each 1-tablespoon/day increment of corn oil, canola oil, or olive oil was associated with 3%, 5%, and 5% lower all-cause mortality, respectively. Similarly, each 1-tablespoon/day increment of corn oil, canola oil, or olive oil was associated with 4%, 6%, and 7% lower cardiometabolic mortality, respectively. Regarding the cause-specific mortality, replacing with 1 tablespoon/day canola oil was associated with lower CVD mortality (6%) and RD mortality (9%), respectively, and replacing with olive oil was related to lower mortality from CVD (6%), RD (14%), AD (25%), and diabetes (13%), respectively. Each 1-tablespoon/day increment of corn oil was related to 24% lower AD mortality (Fig. 3 and Additional file 1: Table S7).

### Subgroup analyses

In secondary analyses for stick and tub/soft margarine consumption, most of the associations were similar except a significant association of stick but not tub/soft margarine consumption with higher AD mortality (Additional file 1: Table S8). Subgroup analyses showed significant modifications for the associations with all-cause mortality stratified by sex, baseline BMI, smoking status, alcohol drinking, income level, and HEI score (Additional file 1: Table S9). Although positive associations of butter and margarine consumption with all-cause mortality persisted in all the subgroups, the associations for butter consumption were stronger among men than women (*P*-interaction = 0.022) and among non-obese participants than obese participants (*P*-interaction< 0.001), while the associations for margarine consumption were more pronounced among non-smokers or former smokers (*P*-interaction = 0.004) and those with higher income level (*P*-interaction< 0.001) and higher HEI score (*P*-interaction = 0.001). Notably, the inverse association of olive oil consumption with all-cause mortality was restricted to alcohol drinkers (*P*-interaction = 0.003).

### Sensitivity analyses

Both significant and non-significant associations did not change materially after exclusion of participants with extreme BMIs, or further adjustment for a propensity score, history of hypertension and hypercholesteremia, or the use of multivitamins and aspirin (Additional file 1: Tables S10-S13). We also detected largely similar associations when we further adjusted for the use of cholesterol-lowering medications; excluded those with CVD, cancer, or diabetes at baseline and those with the first 4 years of follow-up; or censored participants at 8-year follow-up (Additional file 1: Tables S14-S17).

## Discussion

In this large prospective cohort, intakes of butter and margarine were associated with higher all-cause, cardiometabolic, and other major cause-specific mortality, whereas intakes of canola oil and olive oil were related to lower mortality. Substituting corn oil, canola oil, or olive oil for butter and margarine was associated with lower all-cause, cardiometabolic, cancer, RD, and AD mortality.

Current dietary recommendations on butter consumption largely depend on the assumed negative effect of high SFA content (> 65%) [19] in relation to higher all-cause mortality and CVD incidence [20, 21] regardless of beneficial ingredients such as vitamins, PUFAs, MUFAs, and ruminant *trans*-fat. However, a previous multi-center nationwide study reported no significant association of butter consumption with all-cause mortality and CVD incidence [22]. Nonetheless, evidence from intervention trials indicated that the butter-enriched diet elevated total and LDL cholesterol compared with the diet rich in vegetable oils or coconut oil [5, 6]. In the current US prospective study, we observed strong associations with higher total and CVD mortality. These data emphasize the reductions in butter consumption among the US population for the management of cardiovascular health. Interestingly, a previous meta-analysis of 11 studies revealed a weak inverse relationship of butter with the risk of diabetes [9], whereas we found participants consuming > 8.6 g 2000 kcal^−1^ day^−1^ had an 18% higher risk of diabetes mortality compared with non-consumers in our study. In accordance with our finding, replacing butter with olive oil was related to a lower risk of diabetes among US women [17]. Our finding of butter consumption in relation to higher cancer mortality was supported by previous studies showing higher incidence of breast cancer and non-Hodgkin lymphoma [23, 24], whereas the other positive associations were first reported. Together, our documented detrimental effects of butter on mortality supported the current US dietary recommendation on decreasing butter intake and highlighted the need for further mechanistic studies on the metabolic effects of butter. In addition, we found higher all-cause and RD mortality among lard consumers. Despite the absent evidence in humans, lard has been frequently used as a high-fat diet to induce obese phenotype and metabolic dysfunction in rodents [25]. More investigations are warranted given the low consumption of lard in the current study.

Margarine contains *trans*-fat, a well-documented risk factor for arterial calcification and coronary heart disease [26], and has a negative impact on plasma lipid profiles in both healthy individuals and patients with hypercholesterolemia [27]. Our findings were consistent with a recent meta-analysis, showing a positive association of *trans*-fat with all-cause and CVD mortality [28]. The observed association of margarine intake with modestly higher diabetes mortality was in line with a European multi-center study [29]. In addition, higher incidence of asthma onset was contributed by the intake of margarine [30], supporting our finding of elevated RD mortality. Compared with tub/soft margarine, our secondary analysis showed that stick margarine consumption turned to be much stronger for its positive association with AD mortality, which could be explained by higher *trans*-fat content (15–21%) [31] and supported by previous evidence suggesting a negative effect of *trans*-fat on dementia [32]. Taken together, our results suggest the importance of restricting intake of *trans*-fat containing margarines to decrease the incidence of cardiometabolic diseases.

We observed overall neutral associations of corn oil consumption with mortality only except a marginal inverse association with stroke mortality. Corn oil could ameliorate plasma atherogenic lipids among participants with elevated cholesterol [33]. However, substituting vegetable oils rich in linoleic acid for SFAs [34] was not associated with lower all-cause or CVD mortality [35]. Unlike corn oil, canola oil as a cardioprotective contributor is rich in MUFAs and α-linolenic acid [36]. Canola oil supplementation could ameliorate overall blood lipid profiles and improve glycemic control and inflammation [6]. Consistently, we showed a protective relationship between canola oil consumption and all-cause and heart disease mortality. The observed lower RD and infection mortality might be ascribed to immunomodulatory and anti-inflammatory effects of MUFAs [37].

The cardioprotective association of olive oil has been supported by the Spanish European Prospective into Cancer and Nutrition study [38], the Nurses’ Health Study and the Health Professionals Follow-up Study [39], and the PREvención con DIeta MEDiterránea study of patients at high CVD incidence [40]. Consistent with our finding, a previous US women study also showed a lower risk of diabetes with higher olive oil intake [17]. Mediterranean diet supplemented with olive oil seemed to be effective in reducing the risk of developing diabetes among participants at high cardiovascular risk [41]. A recent meta-analysis also demonstrated that olive oil supplementation could lower HbA1c and fasting plasma glucose levels among diabetic patients [8]. Besides, the association with lower AD mortality might be due to the neuroprotective phenols and oleocanthal in olive oil. Collectively, these data underscored the health benefits of olive oil and provided evidence on habitual olive oil consumption as a key contributor to the healthy Mediterranean diet [40]. Increasing the consumption of olive oil may confer health benefits on cardiometabolic health and reduce mortality.

Subgroup analyses demonstrated stronger associations of butter consumption with higher mortality among men than women, which could be due to sex-dependent fatty acid metabolism [42], and higher CVD mortality rates and lower life expectancy in men than women. The interaction between olive oil and alcohol drinking status on mortality might be due to the correlation between olive oil consumption and the Mediterranean pattern which included wine. Future research is warranted to elucidate the observed interactions between smoking, alcohol drinking, BMI, and HEI score and cooking oils/fats.

Strengths of the current study include the large population size, long-term duration with a high follow-up rate (> 99%), and a large number of deaths from various causes. We excluded participants with chronic diseases at baseline or initial 4 years of follow-up to further reduce the possibility of reverse causality and found similar results, indicating the robustness of our findings. The limitations should also be noted. First, the observed associations might be partly due to residual confounding despite comprehensive adjustment for well-known risk factors. Second, although validated, our FFQ might still produce measurement errors. Given the prospective study design, any mismeasurement in cooking oil/fat consumption would likely be random for mortality, resulting in conservative associations. Third, the overall intake level of individual vegetable oils was low in this population with a relatively narrow intake range. Nonetheless, we still detected significant protective associations of these vegetable oils, especially when substituting for solid fats. Fourth, due to the lack of measurement data, we could not further analyze the associations between fats/oils with different cooking methods and mortality, which could make our results more informative. In addition, dietary intakes were only assessed at baseline, and potential dietary changes could occur during the long-term follow-up. Nonetheless, this might not appreciably change our results because we also observed similar associations when censoring participants at a shorter duration (8 years) of follow-up. Moreover, the potential changes in the constituents of specific oils could not be captured. Typically, margarines contain high amount of *trans-*fat in 1990s. However, *trans*-fat was phased out in the last decade. In recent years, soft margarines have contained no *trans*-fat and only hard and tub may still contain. This probably would have weakened the observed positive associations of margarine consumption. Finally, a causal relationship may not be established due to the observational nature.

## Conclusion

In summary, consumption of butter and margarine was associated with higher all-cause and cardiometabolic mortality. Intakes of canola oil and olive oil were associated with lower total mortality and corn oil had a neutral association with mortality. From the standpoint of public health, intakes of butter and margarine may be limited while olive oil consumption may be recommended to lower deaths from cardiometabolic diseases. Replacing butter and margarine with corn oil, canola oil, or olive oil may confer health benefits on cardiometabolic health and reduce mortality. Taken together, current dietary recommendations should continue to highlight shifting the intake from solid fats, including butter and margarine, to non-hydrogenated vegetable oils, such as corn oil, canola oil, and olive oil, for the prevention of cardiometabolic diseases and premature deaths.

## Supplementary Information

**Additional file 1: Figure S1.** Flow of participants in current NIH-AARP prospective cohort. **Table S1.** Categories for causes of death. **Table S2.** Baseline characteristics of participants according to corn oil, canola oil and olive oil consumption. **Table S3.** Spearman correlations between individual cooking oils. **Table S4.** Multivariable-adjusted HRs (95% CIs) of heart disease and stroke mortality according to individual oil consumption. **Table S5.** Multivariable-adjusted HRs (95% CIs) of mortality from non-cardiometabolic causes according to individual oil consumption. **Table S6.** Multivariable-adjusted HRs (95% CIs) of all-cause and cause-specific mortality according to lard consumption. **Table S7.** Data source of Fig. 3. Multivariable-adjusted HRs (95% CIs) for substituting tablespoon/d canola oil, corn oil, or olive oil for equivalent amounts of butter and margarine. **Table S8.** Multivariable-adjusted HRs (95% CIs) of all-cause and cause-specific mortality associated with consumption of stick margarine and other margarine. **Table S9.** Multivariable-adjusted HRs (95% CIs) of all-cause mortality from subgroup analyses. **Table S10.** Multivariable-adjusted HRs (95% CIs) of all-cause and cause-specific mortality from the sensitivity analysis that excluding those with extreme BMIs. **Table S11.** Multivariable-adjusted HRs (95% CIs) of all-cause and cause-specific mortality from the sensitivity analysis that further adjusting for a propensity score. **Table S12.** Multivariable-adjusted HRs (95% CIs) of all-cause and cause-specific mortality from the sensitivity analysis that further adjusted for history of hypertension and hypercholesteremia. **Table S13.** Multivariable-adjusted HRs (95% CIs) of all-cause and cause-specific mortality from the sensitivity analysis that further adjusted for aspirin and multivitamins use. **Table S14.** Multivariable-adjusted HRs (95% CIs) of all-cause and cause-specific mortality from the sensitivity analysis that further adjusted for the use of cholesterol-lowering medications (*n* = 293,918). **Table S15.** Multivariable-adjusted HRs (95% CIs) of all-cause and cause-specific mortality from the sensitivity analysis that excluding those with cardiovascular disease, cancer, or diabetes at baseline. **Table S16.** Multivariable-adjusted HRs (95% CIs) of all-cause and cause-specific mortality from the sensitivity analysis that excluding the first 4 years of follow-up. **Table S17.** Multivariable-adjusted HRs (95% CIs) of all-cause and cause-specific mortality from the sensitivity analysis that followed up for 8 years.

## Data Availability

Because of the sensitive nature of the data collected for this study, requests to access the dataset from qualified researchers trained in human subject confidentiality protocols may be sent to the National Cancer Institute Division of Cancer Epidemiology & Genetics to Linda M. Liao (e-mail: liaolm@mail.nih.gov).

## Abbreviations

AARP: Formerly known as the American Association for Retired Persons
AD: Alzheimer’s disease
BMI: Body mass index
CI: Confidence interval
CVD: Cardiovascular disease
FFQ: Food frequency questionnaire
HEI: Healthy Eating Index
HR: Hazard ratio
MUFA: Monounsaturated fatty acid
PUFA: Polyunsaturated fatty acid
RD: Respiratory disease
SFA: Saturated fatty acid
