# Supplementary material for: Cooking oil/fat consumption and deaths from cardiometabolic diseases and other causes: prospective analysis of 521,120 individuals
Source: BMC Med. 2021 Apr 15;19:92. doi: 10.1186/s12916-021-01961-2 (PMC8048052; doi:10.1186/s12916-021-01961-2)
Supplement: Supplementary file 1 — Additional file 1: Figure S1. Flow of participants in current NIH-AARP prospective cohort. Table S1. Categories for causes of death. Table S2. Baseline characteristics of participants according to corn oil, canola oil and olive oil consumption. Table S3. Spearman correlations between individual cooking oils. Table S4. Multivariable-adjusted HRs (95% CIs) of heart disease and stroke mortality according to individual oil consumption. Table S5. Multivariable-adjusted HRs (95% CIs) of mortality from non-cardiometabolic causes according to individual oil consumption. Table S6. Multivariable-adjusted HRs (95% CIs) of all-cause and cause-specific mortality according to lard consumption. Table S7. Data source of Fig. 3. Multivariable-adjusted HRs (95% CIs) for substituting tablespoon/d canola oil, corn oil, or olive oil for equivalent amounts of butter and margarine. Table S8. Multivariable-adjusted HRs (95% CIs) of all-cause and cause-specific mortality associated with consumption of stick margarine and other margarine. Table S9. Multivariable-adjusted HRs (95% CIs) of all-cause mortality from subgroup analyses. Table S10. Multivariable-adjusted HRs (95% CIs) of all-cause and cause-specific mortality from the sensitivity analysis that excluding those with extreme BMIs. Table S11. Multivariable-adjusted HRs (95% CIs) of all-cause and cause-specific mortality from the sensitivity analysis that further adjusting for a propensity score. Table S12. Multivariable-adjusted HRs (95% CIs) of all-cause and cause-specific mortality from the sensitivity analysis that further adjusted for history of hypertension and hypercholesteremia. Table S13. Multivariable-adjusted HRs (95% CIs) of all-cause and cause-specific mortality from the sensitivity analysis that further adjusted for aspirin and multivitamins use. Table S14. Multivariable-adjusted HRs (95% CIs) of all-cause and cause-specific mortality from the sensitivity analysis that further adjusted for the use of cholesterol-lowering m [file 12916_2021_1961_MOESM1_ESM.docx]

**ONLINE SUPPLEMENTAL MATERIAL**

**Table of Contents**

[Figure S1. Flow of participants in current NIH-AARP prospective cohort 2](#_Toc55382527)

[Table S1. Categories for causes of death 3](#_Toc55382528)

[Table S2. Baseline characteristics of participants according to corn oil, canola oil and olive oil consumption 4](#_Toc55382529)

[Table S3. Spearman correlations between individual cooking oils 6](#_Toc55382530)

[Table S4. Multivariable-adjusted HRs (95% CIs) of heart disease and stroke mortality according to individual oil consumption 7](#_Toc55382531)

[Table S5. Multivariable-adjusted HRs (95% CIs) of mortality from non-cardiometabolic causes according to individual oil consumption 9](#_Toc55382532)

[Table S6. Multivariable-adjusted HRs (95% CIs) of all-cause and cause-specific mortality according to lard consumption 15](#_Toc55382533)

[Table S7. Data source of figure 3. Multivariable-adjusted HRs (95% CIs) for substituting 1 tablespoon/d canola oil, corn oil, or olive oil for equivalent amounts of butter and margarine 16](#_Toc55382534)

[Table S8. Multivariable-adjusted HRs (95% CIs) of all-cause and cause-specific mortality associated with consumption of stick margarine and other margarine 17](#_Toc55382535)

[Table S9. Multivariable-adjusted HRs (95% CIs) of all-cause mortality from subgroup analyses 19](#_Toc55382536)

[Table S10. Multivariable-adjusted HRs (95% CIs) of all-cause and cause-specific mortality from the sensitivity analysis that excluding those with extreme BMIs 22](#_Toc55382537)

[Table S11. Multivariable-adjusted HRs (95% CIs) of all-cause and cause-specific mortality from the sensitivity analysis that further adjusting for a propensity score 25](#_Toc55382538)

[Table S12. Multivariable-adjusted HRs (95% CIs) of all-cause and cause-specific mortality from the sensitivity analysis that further adjusted for history of hypertension and hypercholesteremia 28](#_Toc55382539)

[Table S13. Multivariable-adjusted HRs (95% CIs) of all-cause and cause-specific mortality from the sensitivity analysis that further adjusted for aspirin and multivitamins use 31](#_Toc55382540)

[Table S14. Multivariable-adjusted HRs (95% CIs) of all-cause and cause-specific mortality from the sensitivity analysis that further adjusted for the use of cholesterol-lowering medications (n=293,918) 34](#_Toc55382541)

[Table S15. Multivariable-adjusted HRs (95% CIs) of all-cause and cause-specific mortality from the sensitivity analysis that excluding those with cardiovascular disease, cancer, or diabetes at baseline 37](#_Toc55382542)

[Table S16. Multivariable-adjusted HRs (95% CIs) of all-cause and cause-specific mortality from the sensitivity analysis that excluding the first 4 years of follow-up 40](#_Toc55382543)

[Table S17. Multivariable-adjusted HRs (95% CIs) of all-cause and cause-specific mortality from the sensitivity analysis that followed up for 8 years 43](#_Toc55382544)


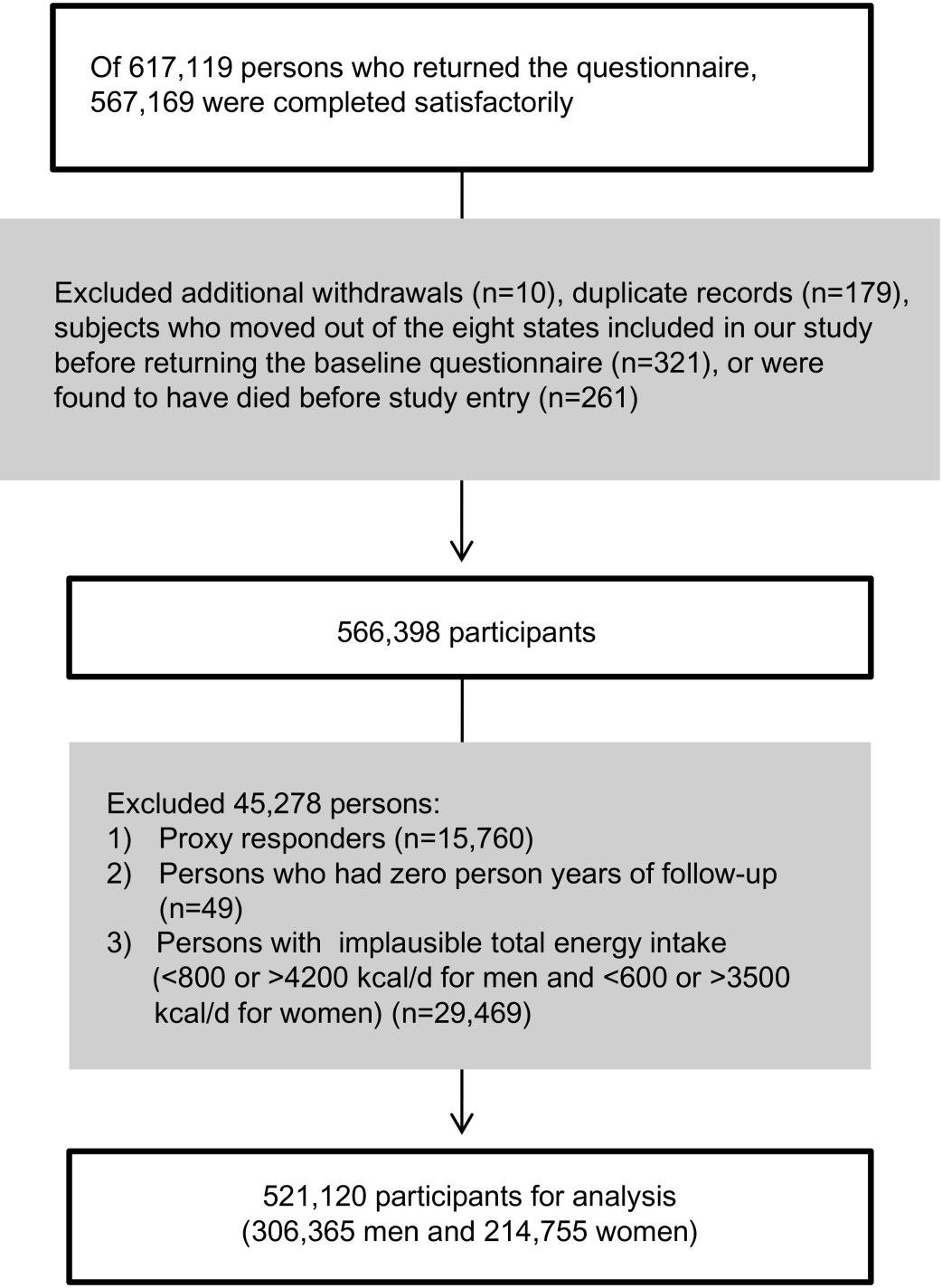


# Figure S1. Flow of participants in current NIH-AARP prospective cohort

# Table S1. Categories for causes of death

| Causes of Death | ICD-9 Code | ICD-10 Code |
| --- | --- | --- |
| Cancer | 140-239 | C00-C97 and D00-D48 |
| Alzheimer's disease | 331 | G30 |
| Cardiovascular disease | 390-398, 401-404, 410-429 and 440-448 | I00-I13, I20-I51 and I70-I78 |
| Diabetes | 250 | E10-E14 |
| Respiratory disease | 480-487 and 490-496 | J10-J18 and J40-J47 |
| Infections | 001-139 | A00-B99 |
| Kidney disease | 580-589 | N00-N07, N17-N19 and N25-N27 |
| Chronic liver disease | 571 | K70, K73-K74 |
| Injuries and accidents | 800-978 | V01-X59, Y85-Y86, U03, X60-X84, Y87.0, U01-U02, X85-Y09, Y35, Y87.1, and Y89.0 |

Detailed corresponding ICD codes for these categories are available at https://seer.cancer.gov/codrecode/1969+_d09172004/index.html.

# Table S2. Baseline characteristics of participants according to corn oil, canola oil and olive oil consumption

|  | Corn oil consumption | | | |  | Canola oil consumption | | | |  | Olive oil consumption | | | |
| --- | --- | --- | --- | --- | --- | --- | --- | --- | --- | --- | --- | --- | --- | --- |
| Characteristics | Non-  consumers | T1 | T2 | T3 |  | Non-  consumers | T1 | T2 | T3 |  | Non-  consumers | T1 | T2 | T3 |
| Range (g·2000 kcal^-1^·d^-1^) | 0 | ≤0.6 | 0.7-1.7 | ≥1.8 |  | 0 | ≤0.6 | 0.7-1.6 | ≥1.7 |  | 0 | ≤0.7 | 0.8-1.8 | ≥1.9 |
| N | 399,360 | 40,586 | 40,587 | 40,587 |  | 376,913 | 48,069 | 48,069 | 48,069 |  | 353,766 | 55,784 | 55,785 | 55,785 |
| Age (y) | 62.8 | 62.4 | 62.9 | 63.2 |  | 62.8 | 62.5 | 63.0 | 63.3 |  | 63.0 | 62.1 | 62.4 | 62.7 |
| Male (%) | 58.9 | 58.1 | 58.9 | 58.5 |  | 60.6 | 53.9 | 53.3 | 54.9 |  | 60.1 | 53.3 | 55.7 | 59.5 |
| Race (%) |  |  |  |  |  |  |  |  |  |  |  |  |  |  |
| White | 92.9 | 92.2 | 89.4 | 82.4 |  | 91.7 | 93.9 | 92.8 | 89.0 |  | 91.1 | 94.6 | 93.6 | 91.2 |
| Black | 2.9 | 4.1 | 5.9 | 8.6 |  | 3.7 | 2.7 | 3.2 | 4.3 |  | 4.3 | 2.0 | 2.2 | 2.3 |
| Hispanic | 1.6 | 1.5 | 1.9 | 3.5 |  | 1.8 | 1.3 | 1.5 | 2.4 |  | 1.6 | 1.5 | 2.0 | 3.1 |
| Asian | 1.0 | 0.9 | 1.3 | 3.4 |  | 1.0 | 0.9 | 1.1 | 2.7 |  | 1.2 | 0.7 | 0.9 | 1.8 |
| BMI (kg/m^2^) | 26.3 | 26.6 | 26.6 | 26.6 |  | 26.5 | 26.3 | 26.1 | 26.4 |  | 26.5 | 26.2 | 26.1 | 26.2 |
| Married (%) | 68.2 | 69.4 | 69.0 | 68.7 |  | 67.4 | 71.0 | 70.4 | 71.6 |  | 67.8 | 69.3 | 69.2 | 70.4 |
| Annual household income (USD)^a^ | 49,511 | 48,975 | 46,652 | 44,111 |  | 48,913 | 50,192 | 48,313 | 46,489 |  | 47,135 | 51,991 | 51,657 | 51,464 |
| College graduate or postgraduate (%) | 40.1 | 41.0 | 35.6 | 30.2 |  | 38.8 | 43.4 | 39.7 | 35.7 |  | 36.6 | 45.7 | 44.1 | 42.4 |
| Current smoker (%) | 11.1 | 11.9 | 13.6 | 15.4 |  | 12.4 | 9.1 | 9.7 | 10.8 |  | 12.3 | 9.8 | 10.5 | 10.9 |
| Physical activity, ≥5 times/wk (%) | 20.0 | 17.8 | 15.6 | 15.2 |  | 19.2 | 20.1 | 19.0 | 17.9 |  | 19.0 | 19.8 | 19.2 | 19.0 |
| Heart disease (%) | 14.3 | 12.7 | 12.9 | 13.5 |  | 13.7 | 14.1 | 14.6 | 15.7 |  | 14.4 | 12.7 | 12.9 | 13.6 |
| Stroke (%) | 2.1 | 1.9 | 2.0 | 2.3 |  | 2.1 | 1.9 | 2.0 | 2.3 |  | 2.3 | 1.7 | 1.7 | 1.9 |
| Cancer (%) | 9.0 | 9.1 | 9.0 | 8.7 |  | 8.9 | 9.4 | 9.3 | 9.4 |  | 9.0 | 9.3 | 9.0 | 8.8 |
| Diabetes (%) | 9.0 | 8.2 | 9.1 | 10.9 |  | 9.2 | 7.9 | 8.5 | 10.4 |  | 9.8 | 7.1 | 7.4 | 8.5 |
| Fair or poor health (%) | 12.4 | 12.1 | 13.9 | 17.0 |  | 13.0 | 11.0 | 11.9 | 15.0 |  | 13.8 | 10.4 | 10.5 | 12.0 |
| Currently uses multivitamins (%) | 56.5 | 56.4 | 53.3 | 49.7 |  | 54.5 | 60.8 | 59.0 | 56.3 |  | 54.3 | 60.6 | 58.8 | 56.6 |
| Daily use of aspirin (%) | 15.2 | 15.2 | 13.6 | 12.9 |  | 14.5 | 16.7 | 15.9 | 15.3 |  | 14.9 | 15.8 | 14.9 | 14.2 |
| Daily dietary intake |  |  |  |  |  |  |  |  |  |  |  |  |  |  |
| Total energy (kcal/d) | 1663.5 | 1858.8 | 1771.4 | 1676.8 |  | 1684.5 | 1805.3 | 1656.9 | 1613.7 |  | 1676.9 | 1797.8 | 1695.6 | 1631.4 |
| Alcohol from alcoholic drinks (g/d) | 1.9 | 2.3 | 1.8 | 1.1 |  | 1.8 | 2.4 | 1.9 | 1.4 |  | 1.4 | 3.5 | 3.6 | 3.6 |
| Total protein (% of energy) | 15.4 | 15.3 | 15.1 | 14.6 |  | 15.3 | 15.5 | 15.4 | 15.1 |  | 15.4 | 15.5 | 15.3 | 14.9 |
| Total fat (% of energy) | 29.7 | 30.6 | 32.3 | 34.6 |  | 30.4 | 28.8 | 29.8 | 32.3 |  | 30.5 | 28.8 | 29.6 | 31.7 |
| Healthy Eating Index score | 69.2 | 68.1 | 66.8 | 66.1 |  | 68.1 | 70.6 | 70.3 | 70.0 |  | 68.0 | 70.3 | 69.9 | 69.8 |

Data are medians or percentages. BMI, body mass index; T, tertile.

^a^ Household income in 1999.

# Table S3. Spearman correlations between individual cooking oils

|  | Butter | Margarine | Canola oil | Corn oil | Olive oil |
| --- | --- | --- | --- | --- | --- |
| Butter | 1.00 | -0.43 | -0.05 | -0.01 | 0.11 |
| Margarine |  | 1.00 | 0.05 | 0.06 | -0.09 |
| Canola oil |  |  | 1.00 | -0.04 | 0.07 |
| Corn oil |  |  |  | 1.00 | 0.00 |
| Olive oil |  |  |  |  | 1.00 |

# Table S4. Multivariable-adjusted HRs (95% CIs) of heart disease and stroke mortality according to individual oil consumption

|  | Categories of individual oil consumption | | | |  |
| --- | --- | --- | --- | --- | --- |
|  | Non-consumers | T1 | T2 | T3 | *P* trend |
| Heart disease |  |  |  |  |  |
| Butter |  |  |  |  |  |
| Death cases/*n* | 20,024/303,987 | 3,960/72,377 | 4,491/72,378 | 4,667/72,378 |  |
| Multivariable-adjusted HR (95% CI) | 1.00 | 0.96 (0.93-1.00) | 1.05 (1.01-1.09) | 1.08 (1.04-1.12) | <0.001 |
| Margarine |  |  |  |  |  |
| Death cases/*n* | 7,889/134,374 | 7,430/128,915 | 8,521/128,916 | 9,302/128,915 |  |
| Multivariable-adjusted HR (95% CI) | 1.00 | 1.01 (0.98-1.05) | 1.06 (1.02-1.10) | 1.10 (1.06-1.14) | <0.001 |
| Corn oil |  |  |  |  |  |
| Death cases/*n* | 25,114/399,360 | 2,446/40,586 | 2,626/40,587 | 2,956/40,587 |  |
| Multivariable-adjusted HR (95% CI) | 1.00 | 1.02 (0.98-1.07) | 0.99 (0.95-1.03) | 1.01 (0.97-1.05) | 0.65 |
| Canola oil |  |  |  |  |  |
| Death cases/*n* | 24,434/376,913 | 2,682/48,069 | 2,860/48,069 | 3,166/48,069 |  |
| Multivariable-adjusted HR (95% CI) | 1.00 | 0.99 (0.95-1.04) | 0.98 (0.94-1.02) | 0.96 (0.93-1.00) | 0.041 |
| Olive oil |  |  |  |  |  |
| Death cases/*n* | 23,916/353,766 | 2,869/55,784 | 3,070/55,785 | 3,287/55,785 |  |
| Multivariable-adjusted HR (95% CI) | 1.00 | 0.92 (0.88-0.97) | 0.96 (0.93-1.00) | 0.96 (0.92-0.99) | 0.009 |
| Stroke |  |  |  |  |  |
| Butter |  |  |  |  |  |
| Death cases/*n* | 3,382/303,987 | 663/72,377 | 722/72,378 | 838/72,378 |  |
| Multivariable-adjusted HR (95% CI) | 1.00 | 0.96 (0.88-1.06) | 1.00 (0.92-1.09) | 1.09 (0.99-1.19) | 0.059 |
| Margarine |  |  |  |  |  |
| Death cases/*n* | 1,416/134,374 | 1,200/128,915 | 1,380/128,916 | 1,609/128,915 |  |
| Multivariable-adjusted HR (95% CI) | 1.00 | 0.95 (0.87-1.04) | 1.02 (0.94-1.11) | 1.10 (1.00-1.20) | 0.003 |
| Corn oil |  |  |  |  |  |
| Death cases/*n* | 4,329/399,360 | 384/40,586 | 442/40,587 | 450/40,587 |  |
| Multivariable-adjusted HR (95% CI) | 1.00 | 0.91 (0.81-1.02) | 0.99 (0.89-1.09) | 0.91 (0.82-1.01) | 0.061 |
| Canola oil |  |  |  |  |  |
| Death cases/*n* | 4,086/376,913 | 467/48,069 | 502/48,069 | 550/48,069 |  |
| Multivariable-adjusted HR (95% CI) | 1.00 | 0.99 (0.88-1.11) | 0.99 (0.89-1.09) | 0.99 (0.91-1.09) | 0.82 |
| Olive oil |  |  |  |  |  |
| Death cases/*n* | 4,046/353,766 | 508/55,784 | 508/55,785 | 543/55,785 |  |
| Multivariable-adjusted HR (95% CI) | 1.00 | 0.95 (0.85-1.06) | 0.89 (0.81-0.99) | 0.92 (0.84-1.00) | 0.026 |

CI, confidence interval; HR, hazard ratio; T, tertile. HRs were adjusted for age, sex, BMI, race, education, marital status, household income, smoking, alcohol, vigorous physical activity, usual activity at work, perceived health condition, history of heart disease, stroke, diabetes, and cancer at baseline, Healthy Eating Index-2015, total energy intake, and consumption of remaining oils where appropriate (butter, margarine, lard, corn oil, canola oil, olive oil, and other vegetable oils).

# Table S5. Multivariable-adjusted HRs (95% CIs) of mortality from non-cardiometabolic causes according to individual oil consumption

|  | Categories of individual oil consumption | | | |  |
| --- | --- | --- | --- | --- | --- |
|  | Non-consumers | T1 | T2 | T3 | *P* trend |
| Cancer |  |  |  |  |  |
| Butter |  |  |  |  |  |
| Death cases/*n* | 26,262/303,987 | 5,735/72,377 | 6,563/72,378 | 7,223/72,378 |  |
| Multivariable-adjusted HR (95% CI) | 1.00 | 0.98 (0.95-1.01) | 1.03 (1.00-1.07) | 1.06 (1.03-1.09) | <0.001 |
| Margarine |  |  |  |  |  |
| Death cases/*n* | 12,054/134,374 | 10,495/128,915 | 11,532/128,916 | 11,702/128,915 |  |
| Multivariable-adjusted HR (95% CI) | 1.00 | 0.98 (0.95-1.01) | 1.00 (0.97-1.03) | 1.01 (0.98-1.05) | 0.061 |
| Corn oil |  |  |  |  |  |
| Death cases/*n* | 34,996/399,360 | 3,389/40,586 | 3,590/40,587 | 3,808/40,587 |  |
| Multivariable-adjusted HR (95% CI) | 1.00 | 0.94 (0.91-0.98) | 0.96 (0.93-1.00) | 0.98 (0.95-1.01) | 0.081 |
| Canola oil |  |  |  |  |  |
| Death cases/*n* | 33,667/376,913 | 3,874/48,069 | 3,954/48,069 | 4,288/48,069 |  |
| Multivariable-adjusted HR (95% CI) | 1.00 | 0.97 (0.94-1.01) | 0.97 (0.94-1.00) | 1.00 (0.97-1.03) | 0.74 |
| Olive oil |  |  |  |  |  |
| Death cases/*n* | 31,604/353,766 | 4,570/55,784 | 4,673/55,785 | 4,936/55,785 |  |
| Multivariable-adjusted HR (95% CI) | 1.00 | 1.03 (0.99-1.07) | 1.01 (0.98-1.05) | 1.02 (0.99-1.05) | 0.16 |
| Respiratory disease |  |  |  |  |  |
| Butter |  |  |  |  |  |
| Death cases/*n* | 5,598/303,987 | 1,045/72,377 | 1,370/72,378 | 1,786/72,378 |  |
| Multivariable-adjusted HR (95% CI) | 1.00 | 1.00 (0.93-1.07) | 1.11 (1.04-1.18) | 1.23 (1.15-1.31) | <0.001 |
| Margarine |  |  |  |  |  |
| Death cases/*n* | 2,555/134,374 | 1,921/128,915 | 2,427/128,916 | 2,896/128,915 |  |
| Multivariable-adjusted HR (95% CI) | 1.00 | 0.98 (0.91-1.05) | 1.09 (1.02-1.17) | 1.23 (1.15-1.31) | <0.001 |
| Corn oil |  |  |  |  |  |
| Death cases/*n* | 7,346/399,360 | 695/40,586 | 797/40,587 | 961/40,587 |  |
| Multivariable-adjusted HR (95% CI) | 1.00 | 0.97 (0.89-1.06) | 0.98 (0.91-1.06) | 1.01 (0.95-1.09) | 0.80 |
| Canola oil |  |  |  |  |  |
| Death cases/*n* | 7,380/376,913 | 737/48,069 | 776/48,069 | 906/48,069 |  |
| Multivariable-adjusted HR (95% CI) | 1.00 | 0.97 (0.89-1.06) | 0.93 (0.86-1.01) | 0.94 (0.86-1.00) | 0.041 |
| Olive oil |  |  |  |  |  |
| Death cases/*n* | 7197/353,766 | 824/55,784 | 848/55,785 | 930/55,785 |  |
| Multivariable-adjusted HR (95% CI) | 1.00 | 0.90 (0.83-0.98) | 0.89 (0.83-0.96) | 0.89 (0.83-0.96) | <0.001 |
| Alzheimer's disease |  |  |  |  |  |
| Butter |  |  |  |  |  |
| Death cases/*n* | 1,254/303,987 | 265/72,377 | 255/72,378 | 272/72,378 |  |
| Multivariable-adjusted HR (95% CI) | 1.00 | 0.94 (0.81-1.09) | 0.93 (0.80-1.07) | 0.94 (0.81-1.10) | 0.44 |
| Margarine |  |  |  |  |  |
| Death cases/*n* | 483/134,374 | 499/128,915 | 509/128,916 | 555/128,915 |  |
| Multivariable-adjusted HR (95% CI) | 1.00 | 1.09 (0.94-1.26) | 1.07 (0.92-1.23) | 1.05 (0.91-1.22) | 0.86 |
| Corn oil |  |  |  |  |  |
| Death cases/*n* | 1594/399,360 | 148/40,586 | 165/40,587 | 139/40,587 |  |
| Multivariable-adjusted HR (95% CI) | 1.00 | 0.98 (0.81-1.18) | 1.01 (0.85-1.19) | 0.84 (0.70-1.00) | 0.061 |
| Canola oil |  |  |  |  |  |
| Death cases/*n* | 1480/376,913 | 184/48,069 | 201/48,069 | 181/48,069 |  |
| Multivariable-adjusted HR (95% CI) | 1.00 | 1.03 (0.86-1.24) | 1.00 (0.86-1.17) | 0.91 (0.78-1.06) | 0.26 |
| Olive oil |  |  |  |  |  |
| Death cases/*n* | 1502/353,766 | 182/55,784 | 197/55,785 | 165/55,785 |  |
| Multivariable-adjusted HR (95% CI) | 1.00 | 0.81 (0.68-0.98) | 0.87 (0.75-1.02) | 0.75 (0.64-0.89) | <0.001 |
| Infections |  |  |  |  |  |
| Butter |  |  |  |  |  |
| Death cases/*n* | 1491/303,987 | 310/72,377 | 361/72,378 | 407/72,378 |  |
| Multivariable-adjusted HR (95% CI) | 1.00 | 1.02 (0.89-1.17) | 1.07 (0.94-1.21) | 1.11 (0.98-1.27) | 0.094 |
| Margarine |  |  |  |  |  |
| Death cases/*n* | 664/134,374 | 544/128,915 | 666/128,916 | 695/128,915 |  |
| Multivariable-adjusted HR (95% CI) | 1.00 | 0.90 (0.79-1.03) | 1.01 (0.89-1.15) | 0.99 (0.87-1.14) | 0.44 |
| Corn oil |  |  |  |  |  |
| Death cases/*n* | 1893/399,360 | 180/40,586 | 263/40,587 | 233/40,587 |  |
| Multivariable-adjusted HR (95% CI) | 1.00 | 0.98 (0.83-1.17) | 1.29 (1.12-1.47) | 1.00 (0.87-1.15) | 0.49 |
| Canola oil |  |  |  |  |  |
| Death cases/*n* | 1968/376,913 | 171/48,069 | 208/48,069 | 222/48,069 |  |
| Multivariable-adjusted HR (95% CI) | 1.00 | 0.77 (0.64-0.92) | 0.87 (0.74-1.01) | 0.88 (0.76-1.01) | 0.026 |
| Olive oil |  |  |  |  |  |
| Death cases/*n* | 1848/353,766 | 224/55,784 | 242/55,785 | 255/55,785 |  |
| Multivariable-adjusted HR (95% CI) | 1.00 | 1.05 (0.89-1.24) | 0.93 (0.81-1.07) | 0.97 (0.85-1.11) | 0.57 |
| Kidney disease |  |  |  |  |  |
| Butter |  |  |  |  |  |
| Death cases/*n* | 1092/303,987 | 198/72,377 | 245/72,378 | 312/72,378 |  |
| Multivariable-adjusted HR (95% CI) | 1.00 | 0.96 (0.81-1.14) | 1.09 (0.94-1.27) | 1.27 (1.09-1.47) | 0.001 |
| Margarine |  |  |  |  |  |
| Death cases/*n* | 455/134,374 | 372/128,915 | 460/128,916 | 560/128,915 |  |
| Multivariable-adjusted HR (95% CI) | 1.00 | 0.97 (0.83-1.14) | 1.05 (0.90-1.22) | 1.17 (1.00-1.36) | 0.009 |
| Corn oil |  |  |  |  |  |
| Death cases/*n* | 1406/399,360 | 109/40,586 | 136/40,587 | 196/40,587 |  |
| Multivariable-adjusted HR (95% CI) | 1.00 | 0.83 (0.67-1.03) | 0.89 (0.74-1.07) | 1.07 (0.92-1.25) | 0.56 |
| Canola oil |  |  |  |  |  |
| Death cases/*n* | 1388/376,913 | 129/48,069 | 139/48,069 | 191/48,069 |  |
| Multivariable-adjusted HR (95% CI) | 1.00 | 0.96 (0.78-1.18) | 0.86 (0.71-1.03) | 0.97 (0.83-1.13) | 0.51 |
| Olive oil |  |  |  |  |  |
| Death cases/*n* | 1354/353,766 | 136/55,784 | 161/55,785 | 196/55,785 |  |
| Multivariable-adjusted HR (95% CI) | 1.00 | 0.90 (0.73-1.11) | 0.99 (0.83-1.18) | 1.01 (0.86-1.17) | 0.95 |
| Chronic liver disease |  |  |  |  |  |
| Butter |  |  |  |  |  |
| Death cases/*n* | 546/303,987 | 113/72,377 | 179/72,378 | 209/72,378 |  |
| Multivariable-adjusted HR (95% CI) | 1.00 | 0.91 (0.73-1.13) | 1.20 (1.00-1.44) | 1.35 (1.11-1.64) | <0.001 |
| Margarine |  |  |  |  |  |
| Death cases/*n* | 304/134,374 | 219/128,915 | 297/128,916 | 227/128,915 |  |
| Multivariable-adjusted HR (95% CI) | 1.00 | 0.88 (0.72-1.07) | 1.09 (0.90-1.31) | 0.95 (0.77-1.17) | 0.88 |
| Corn oil |  |  |  |  |  |
| Death cases/*n* | 785/399,360 | 75/40,586 | 90/40,587 | 97/40,587 |  |
| Multivariable-adjusted HR (95% CI) | 1.00 | 0.89 (0.68-1.17) | 1.06 (0.84-1.33) | 1.10 (0.88-1.36) | 0.38 |
| Canola oil |  |  |  |  |  |
| Death cases/*n* | 797/376,913 | 72/48,069 | 84/48,069 | 94/48,069 |  |
| Multivariable-adjusted HR (95% CI) | 1.00 | 0.76 (0.57-1.00) | 0.94 (0.74-1.19) | 1.02 (0.82-1.27) | 0.98 |
| Olive oil |  |  |  |  |  |
| Death cases/*n* | 713/353,766 | 111/55,784 | 112/55,785 | 111/55,785 |  |
| Multivariable-adjusted HR (95% CI) | 1.00 | 1.27 (0.99-1.62) | 1.02 (0.82-1.26) | 0.95 (0.77-1.17) | 0.64 |
| Other causes |  |  |  |  |  |
| Butter |  |  |  |  |  |
| Death cases/*n* | 14018/303,987 | 3114/72,377 | 3302/72,378 | 3544/72,378 |  |
| Multivariable-adjusted HR (95% CI) | 1.00 | 1.00 (0.96-1.04) | 1.05 (1.01-1.09) | 1.09 (1.04-1.14) | <0.001 |
| Margarine |  |  |  |  |  |
| Death cases/*n* | 6031/134,374 | 5658/128,915 | 5895/128,916 | 6394/128,915 |  |
| Multivariable-adjusted HR (95% CI) | 1.00 | 1.00 (0.96-1.05) | 1.00 (0.96-1.04) | 1.06 (1.01-1.10) | 0.004 |
| Corn oil |  |  |  |  |  |
| Death cases/*n* | 18366/399,360 | 1739/40,586 | 1835/40,587 | 2038/40,587 |  |
| Multivariable-adjusted HR (95% CI) | 1.00 | 0.97 (0.91-1.02) | 0.95 (0.91-1.00) | 0.99 (0.94-1.03) | 0.33 |
| Canola oil |  |  |  |  |  |
| Death cases/*n* | 17747/376,913 | 1985/48,069 | 2080/48,069 | 2166/48,069 |  |
| Multivariable-adjusted HR (95% CI) | 1.00 | 0.97 (0.92-1.03) | 0.96 (0.92-1.01) | 0.93 (0.89-0.97) | <0.001 |
| Olive oil |  |  |  |  |  |
| Death cases/*n* | 17133/353,766 | 2197/55,784 | 2266/55,785 | 2382/55,785 |  |
| Multivariable-adjusted HR (95% CI) | 1.00 | 0.93 (0.88-0.98) | 0.93 (0.89-0.98) | 0.93 (0.89-0.97) | <0.001 |

CI, confidence interval; HR, hazard ratio; T, tertile. HRs were adjusted for age, sex, BMI, race, education, marital status, household income, smoking, alcohol, vigorous physical activity, usual activity at work, perceived health condition, history of heart disease, stroke, diabetes, and cancer at baseline, Healthy Eating Index-2015, total energy intake, and consumption of remaining oils where appropriate (butter, margarine, lard, corn oil, canola oil, olive oil, and other vegetable oils).

# Table S6. Multivariable-adjusted HRs (95% CIs) of all-cause and cause-specific mortality according to lard consumption

|  | Lard consumption | |  |
| --- | --- | --- | --- |
|  | Non-consumers | Consumers | *P* |
| All-cause |  |  |  |
| Death cases/*n* | 126,206/510,552 | 3122/10,568 |  |
| Multivariable-adjusted HR (95% CI) | 1.00 | 1.04 (1.00-1.08) | 0.048 |
| Cardiovascular disease |  |  |  |
| Death cases/*n* | 37,887/510,552 | 860/10,568 |  |
| Multivariable-adjusted HR (95% CI) | 1.00 | 1.01 (0.94-1.08) | 0.86 |
| Cancer |  |  |  |
| Death cases/*n* | 44,668/510,552 | 1115/10,568 |  |
| Multivariable-adjusted HR (95% CI) | 1.00 | 1.01 (0.95-1.07) | 0.85 |
| Respiratory disease |  |  |  |
| Death cases/*n* | 9,489/510,552 | 310/10,568 |  |
| Multivariable-adjusted HR (95% CI) | 1.00 | 1.13 (1.01-1.27) | 0.039 |
| Alzheimer's disease |  |  |  |
| Death cases/*n* | 2,012/510,552 | 34/10,568 |  |
| Multivariable-adjusted HR (95% CI) | 1.00 | 0.88 (0.63-1.24) | 0.47 |
| Diabetes |  |  |  |
| Death cases/*n* | 3,433/510,552 | 79/10,568 |  |
| Multivariable-adjusted HR (95% CI) | 1.00 | 1.02 (0.82-1.28) | 0.84 |
| Infections |  |  |  |
| Death cases/*n* | 2,510/510,552 | 59/10,568 |  |
| Multivariable-adjusted HR (95% CI) | 1.00 | 0.94 (0.73-1.23) | 0.66 |
| Kidney disease |  |  |  |
| Death cases/*n* | 1,801/510,552 | 46/10,568 |  |
| Multivariable-adjusted HR (95% CI) | 1.00 | 1.08 (0.80-1.45) | 0.62 |
| Chronic liver disease |  |  |  |
| Death cases/*n* | 1,008/510,552 | 39/10,568 |  |
| Multivariable-adjusted HR (95% CI) | 1.00 | 1.28 (0.92-1.77) | 0.14 |
| Other causes |  |  |  |
| Death cases/*n* | 23,398/510,552 | 580/10,568 |  |
| Multivariable-adjusted HR (95% CI) | 1.00 | 1.12 (1.03-1.21) | 0.011 |

CI, confidence interval; HR, hazard ratio. HRs were adjusted for age, sex, BMI, race, education, marital status, household income, smoking, alcohol, vigorous physical activity, usual activity at work, perceived health condition, history of heart disease, stroke, diabetes, and cancer at baseline, Healthy Eating Index-2015, total energy intake, and consumption of butter, margarine, corn oil, canola oil, olive oil, and other vegetable oils.

# Table S7. Data source of figure 3. Multivariable-adjusted HRs (95% CIs) for substituting 1 tablespoon/d canola oil, corn oil, or olive oil for equivalent amounts of butter and margarine

|  | Total mortality | |  | Cardiometabolic mortality | |  | CVD mortality | |  | Diabetes mortality | |
| --- | --- | --- | --- | --- | --- | --- | --- | --- | --- | --- | --- |
|  | HR (95% CI) | *P* |  | HR (95% CI) | *P* |  | HR (95% CI) | *P* |  | HR (95% CI) | *P* |
| Substitution for butter |  |  |  |  |  |  |  |  |  |  |  |
| Margarine | 0.97 (0.96-0.98) | <0.001 |  | 0.99 (0.96-1.01) | 0.29 |  | 0.99 (0.97-1.02) | 0.49 |  | 0.95 (0.88-1.02) | 0.17 |
| Corn oil | 0.95 (0.93-0.98) | <0.001 |  | 0.95 (0.91-0.99) | 0.018 |  | 0.96 (0.92-1.00) | 0.055 |  | 0.88 (0.77-1.02) | 0.084 |
| Canola oil | 0.94 (0.92-0.96) | <0.001 |  | 0.94 (0.90-0.98) | 0.0026 |  | 0.93 (0.89-0.97) | 0.002 |  | 0.98 (0.86-1.11) | 0.73 |
| Olive oil | 0.93 (0.91-0.96) | <0.001 |  | 0.92 (0.89-0.96) | <0.001 |  | 0.93 (0.89-0.97) | <0.001 |  | 0.85 (0.73-0.98) | 0.022 |
| Substitution for margarine |  |  |  |  |  |  |  |  |  |  |  |
| Butter | 1.03 (1.02-1.04) | <0.001 |  | 1.01 (0.99-1.04) | 0.29 |  | 1.01 (0.98-1.03) | 0.49 |  | 1.06 (0.98-1.14) | 0.17 |
| Corn oil | 0.97 (0.95-0.99) | 0.01 |  | 0.96 (0.92-1.00) | 0.036 |  | 0.96 (0.92-1.01) | 0.08 |  | 0.91 (0.80-1.05) | 0.18 |
| Canola oil | 0.95 (0.93-0.97) | <0.001 |  | 0.94 (0.91-0.98) | 0.0058 |  | 0.94 (0.90-0.98) | 0.003 |  | 1.01 (0.90-1.14) | 0.88 |
| Olive oil | 0.95 (0.93-0.97) | <0.001 |  | 0.93 (0.90-0.96) | <0.001 |  | 0.94 (0.90-0.97) | <0.001 |  | 0.87 (0.76-1.00) | 0.049 |
|  |  |  |  |  |  |  |  |  |  |  |  |
|  | Cancer mortality | |  | Respiratory disease mortality | |  | Alzheimer's disease mortality | |  |  |  |
|  | HR (95% CI) | *P* |  | HR (95% CI) | *P* |  | HR (95% CI) | *P* |  |  |  |
| Substitution for butter |  |  |  |  |  |  |  |  |  |  |  |
| Margarine | 0.96 (0.94-0.98) | <0.001 |  | 0.96 (0.92-1.01) | 0.091 |  | 1.10 (0.98-1.23) | 0.1 |  |  |  |
| Corn oil | 0.95 (0.91-0.99) | 0.017 |  | 0.92 (0.85-1.01) | 0.063 |  | 0.80 (0.62-1.03) | 0.084 |  |  |  |
| Canola oil | 0.95 (0.91-0.99) | 0.023 |  | 0.89 (0.82-0.97) | 0.011 |  | 0.91 (0.73-1.14) | 0.43 |  |  |  |
| Olive oil | 1.00 (0.97-1.04) | 0.93 |  | 0.84 (0.77-0.92) | <0.001 |  | 0.79 (0.64-0.99) | 0.038 |  |  |  |
| Substitution for margarine |  |  |  |  |  |  |  |  |  |  |  |
| Butter | 1.04 (1.02-1.07) | <0.001 |  | 1.04 (0.99-1.08) | 0.091 |  | 0.91 (0.81-1.02) | 0.1 |  |  |  |
| Corn oil | 0.97 (0.93-1.01) | 0.19 |  | 0.94 (0.87-1.02) | 0.16 |  | 0.76 (0.59-0.97) | 0.029 |  |  |  |
| Canola oil | 0.98 (0.93-1.02) | 0.23 |  | 0.91 (0.83-0.99) | 0.034 |  | 0.87 (0.70-1.08) | 0.19 |  |  |  |
| Olive oil | 1.02 (0.98-1.07) | 0.13 |  | 0.86 (0.79-0.93) | <0.001 |  | 0.75 (0.61-0.92) | 0.007 |  |  |  |

HRs were adjusted for age, sex, BMI, race, education, marital status, household income, smoking, alcohol, vigorous physical activity, usual activity at work, perceived health condition, history of heart disease, stroke, diabetes, and cancer at baseline, Healthy Eating Index-2015, total energy intake, total oil intake, and consumption of remaining oils where appropriate (butter, margarine, lard, corn oil, canola oil, olive oil, and other vegetable oils).

# Table S8. Multivariable-adjusted HRs (95% CIs) of all-cause and cause-specific mortality associated with consumption of stick margarine and other margarine

|  | Categories of individual oil consumption | | | |  |
| --- | --- | --- | --- | --- | --- |
|  | Non-consumers | T1 | T2 | T3 | *P* trend |
| All-cause |  |  |  |  |  |
| Stick margarine | 1.00 | 0.96 (0.94-0.98) | 1.00 (0.99-1.02) | 1.05 (1.03-1.07) | <0.001 |
| Other margarine | 1.00 | 0.99 (0.97-1.00) | 1.00 (0.98-1.02) | 1.03 (1.01-1.05) | <0.001 |
| Cardiovascular disease |  |  |  |  |  |
| Stick margarine | 1.00 | 1.01 (0.98-1.04) | 1.03 (1.00-1.07) | 1.07 (1.04-1.10) | <0.001 |
| Other margarine | 1.00 | 0.97 (0.93-1.00) | 1.02 (0.98-1.05) | 1.07 (1.03-1.11) | <0.001 |
| Cancer |  |  |  |  |  |
| Stick margarine | 1.00 | 0.97 (0.94-1.01) | 1.00 (0.97-1.03) | 1.02 (0.99-1.05) | 0.081 |
| Other margarine | 1.00 | 0.98 (0.95-1.01) | 0.97 (0.94-1.00) | 0.99 (0.96-1.02) | 0.53 |
| Respiratory disease |  |  |  |  |  |
| Stick margarine | 1.00 | 0.94 (0.88-1.01) | 1.05 (0.98-1.12) | 1.11 (1.05-1.19) | <0.001 |
| Other margarine | 1.00 | 0.87 (0.81-0.94) | 0.98 (0.92-1.05) | 1.09 (1.02-1.16) | 0.001 |
| Alzheimer's disease |  |  |  |  |  |
| Stick margarine | 1.00 | 1.01 (0.88-1.17) | 1.10 (0.96-1.27) | 1.21 (1.05-1.40) | 0.006 |
| Other margarine | 1.00 | 1.18 (1.03-1.35) | 1.00 (0.87-1.16) | 0.98 (0.85-1.13) | 0.46 |
| Diabetes |  |  |  |  |  |
| Stick margarine | 1.00 | 0.99 (0.88-1.12) | 1.02 (0.91-1.13) | 1.15 (1.03-1.29) | 0.011 |
| Other margarine | 1.00 | 0.98 (0.87-1.09) | 1.04 (0.94-1.16) | 1.06 (0.96-1.18) | 0.17 |
| Infections |  |  |  |  |  |
| Stick margarine | 1.00 | 0.98 (0.85-1.12) | 1.01 (0.90-1.15) | 1.01 (0.89-1.15) | 0.77 |
| Other margarine | 1.00 | 0.85 (0.74-0.97) | 0.97 (0.86-1.10) | 0.97 (0.85-1.10) | 0.91 |
| Kidney disease |  |  |  |  |  |
| Stick margarine | 1.00 | 0.82 (0.69-0.98) | 1.09 (0.95-1.26) | 1.12 (0.97-1.31) | 0.031 |
| Other margarine | 1.00 | 0.93 (0.80-1.10) | 1.03 (0.89-1.20) | 1.09 (0.95-1.26) | 0.15 |
| Chronic liver disease |  |  |  |  |  |
| Stick margarine | 1.00 | 0.76 (0.61-0.94) | 0.84 (0.69-1.03) | 0.90 (0.73-1.11) | 0.48 |
| Other margarine | 1.00 | 0.96 (0.79-1.17) | 1.07 (0.89-1.29) | 0.84 (0.68-1.04) | 0.21 |
| Other causes |  |  |  |  |  |
| Stick margarine | 1.00 | 0.98 (0.94-1.02) | 1.00 (0.96-1.04) | 1.04 (1.00-1.09) | 0.038 |
| Other margarine | 1.00 | 0.98 (0.94-1.03) | 0.99 (0.95-1.03) | 1.01 (0.97-1.05) | 0.70 |

HRs were adjusted for age, sex, BMI, race, education, marital status, household income, smoking, alcohol, vigorous physical activity, usual activity at work, perceived health condition, history of heart disease, stroke, diabetes, and cancer at baseline, Healthy Eating Index-2015, total energy intake, and consumption of remaining oils where appropriate (butter, stick margarine, other margarine, lard, corn oil, canola oil, olive oil, and other vegetable oils).

# Table S9. Multivariable-adjusted HRs (95% CIs) of all-cause mortality from subgroup analyses

|  | Baseline age (y) | | | | |  |
| --- | --- | --- | --- | --- | --- | --- |
|  | ≥65 | |  | <65 | | *P* for interaction |
|  | HR (95% CI) | *P* value |  | HR (95% CI) | *P* value |  |
| Butter (14 g·2000 kcal^-1^·d^-1^) | 1.07 (1.05-1.08) | <0.001 |  | 1.08 (1.06-1.10) | <0.001 | 0.15 |
| Margarine (14 g·2000 kcal^-1^·d^-1^) | 1.04 (1.03-1.06) | <0.001 |  | 1.04 (1.03-1.05) | <0.001 | 0.71 |
| Corn oil (8 g·2000 kcal^-1^·d^-1^) | 0.98 (0.95-1.02) | 0.31 |  | 1.00 (0.97-1.04) | 0.83 | 0.087 |
| Canola oil (8 g·2000 kcal^-1^·d^-1^) | 0.98 (0.95-1.01) | 0.29 |  | 0.96 (0.92-0.99) | 0.014 | 0.49 |
| Olive oil (8 g·2000 kcal^-1^·d^-1^) | 0.96 (0.93-0.99) | 0.004 |  | 1.00 (0.97-1.03) | 0.91 | 0.29 |
|  |  |  |  |  |  |  |
|  | Sex | | | | |  |
|  | Men | |  | Women | | *P* for interaction |
|  | HR (95% CI) | *P* value |  | HR (95% CI) | *P* value |  |
| Butter (14 g·2000 kcal^-1^·d^-1^) | 1.09 (1.07-1.11) | <0.001 |  | 1.05 (1.03-1.07) | <0.001 | 0.022 |
| Margarine (14 g·2000 kcal^-1^·d^-1^) | 1.05 (1.04-1.06) | <0.001 |  | 1.04 (1.03-1.05) | <0.001 | 0.72 |
| Corn oil (8 g·2000 kcal^-1^·d^-1^) | 0.99 (0.96-1.02) | 0.33 |  | 1.01 (0.97-1.04) | 0.80 | 0.99 |
| Canola oil (8 g·2000 kcal^-1^·d^-1^) | 0.98 (0.95-1.01) | 0.18 |  | 0.96 (0.93-1.00) | 0.060 | 0.46 |
| Olive oil (8 g·2000 kcal^-1^·d^-1^) | 0.98 (0.96-1.01) | 0.15 |  | 0.96 (0.93-1.00) | 0.036 | 0.49 |
|  |  |  |  |  |  |  |
|  | Baseline BMI | | | | |  |
|  | ≥30 | |  | <30 | | *P* for interaction |
|  | HR (95% CI) | *P* value |  | HR (95% CI) | *P* value |  |
| Butter (14 g·2000 kcal^-1^·d^-1^) | 1.07 (1.05-1.10) | <0.001 |  | 1.07 (1.06-1.09) | <0.001 | <0.001 |
| Margarine (14 g·2000 kcal^-1^·d^-1^) | 1.04 (1.03-1.06) | <0.001 |  | 1.04 (1.03-1.05) | <0.001 | 0.59 |
| Corn oil (8 g·2000 kcal^-1^·d^-1^) | 1.01 (0.97-1.06) | 0.64 |  | 0.99 (0.96-1.01) | 0.33 | 0.89 |
| Canola oil (8 g·2000 kcal^-1^·d^-1^) | 0.99 (0.95-1.04) | 0.68 |  | 0.97 (0.94-0.99) | 0.017 | 0.27 |
| Olive oil (8 g·2000 kcal^-1^·d^-1^) | 0.98 (0.94-1.03) | 0.43 |  | 0.98 (0.95-1.00) | 0.033 | 0.34 |
|  |  |  |  |  |  |  |
|  | Current smoker | | | | |  |
|  | Yes | |  | No | | *P* for interaction |
|  | HR (95% CI) | *P* value |  | HR (95% CI) | *P* value |  |
| Butter (14 g·2000 kcal^-1^·d^-1^) | 1.06 (1.04-1.09) | <0.001 |  | 1.08 (1.06-1.09) | <0.001 | 0.99 |
| Margarine (14 g·2000 kcal^-1^·d^-1^) | 1.02 (1.01-1.04) | 0.015 |  | 1.05 (1.04-1.06) | <0.001 | 0.004 |
| Corn oil (8 g·2000 kcal^-1^·d^-1^) | 1.01 (0.96-1.06) | 0.85 |  | 0.99 (0.96-1.02) | 0.41 | 0.61 |
| Canola oil (8 g·2000 kcal^-1^·d^-1^) | 0.99 (0.93-1.05) | 0.65 |  | 0.97 (0.95-1.00) | 0.022 | 0.99 |
| Olive oil (8 g·2000 kcal^-1^·d^-1^) | 0.97 (0.93-1.02) | 0.28 |  | 0.98 (0.96-1.00) | 0.057 | 0.96 |
|  |  |  |  |  |  |  |
|  | Alcohol drinker | | | | |  |
|  | Yes | |  | No | | *P* for interaction |
|  | HR (95% CI) | *P* value |  | HR (95% CI) | *P* value |  |
| Butter (14 g·2000 kcal^-1^·d^-1^) | 1.07 (1.06-1.09) | <0.001 |  | 1.08 (1.05-1.10) | <0.001 | 0.82 |
| Margarine (14 g·2000 kcal^-1^·d^-1^) | 1.04 (1.03-1.05) | <0.001 |  | 1.04 (1.03-1.05) | <0.001 | 0.10 |
| Corn oil (8 g·2000 kcal^-1^·d^-1^) | 1.00 (0.97-1.03) | 0.79 |  | 0.99 (0.96-1.03) | 0.64 | 0.34 |
| Canola oil (8 g·2000 kcal^-1^·d^-1^) | 0.98 (0.95-1.01) | 0.26 |  | 0.96 (0.93-1.00) | 0.041 | 0.26 |
| Olive oil (8 g·2000 kcal^-1^·d^-1^) | 0.96 (0.94-0.99) | 0.002 |  | 1.03 (0.98-1.07) | 0.23 | 0.003 |
|  |  |  |  |  |  |  |
|  | Fair/poor health | | | | |  |
|  | Yes | |  | No | | *P* for interaction |
|  | HR (95% CI) | *P* value |  | HR (95% CI) | *P* value |  |
| Butter (14 g·2000 kcal^-1^·d^-1^) | 1.08 (1.05-1.10) | <0.001 |  | 1.07 (1.05-1.08) | <0.001 | 0.41 |
| Margarine (14 g·2000 kcal^-1^·d^-1^) | 1.05 (1.04-1.07) | <0.001 |  | 1.04 (1.03-1.05) | <0.001 | 0.78 |
| Corn oil (8 g·2000 kcal^-1^·d^-1^) | 1.00 (0.96-1.04) | 0.89 |  | 0.99 (0.96-1.02) | 0.55 | 0.24 |
| Canola oil (8 g·2000 kcal^-1^·d^-1^) | 0.98 (0.94-1.02) | 0.37 |  | 0.97 (0.94-1.00) | 0.029 | 0.68 |
| Olive oil (8 g·2000 kcal^-1^·d^-1^) | 0.96 (0.92-1.00) | 0.047 |  | 0.98 (0.96-1.01) | 0.11 | 0.78 |
|  |  |  |  |  |  |  |
|  |  |  |  |  |  |  |
|  | Income level | | | | |  |
|  | Below median | |  | Above median | | *P* for interaction |
|  | HR (95% CI) | *P* value |  | HR (95% CI) | *P* value |  |
| Butter (14 g·2000 kcal^-1^·d^-1^) | 1.07 (1.05-1.09) | <0.001 |  | 1.08 (1.06-1.10) | <0.001 | 0.72 |
| Margarine (14 g·2000 kcal^-1^·d^-1^) | 1.04 (1.02-1.05) | <0.001 |  | 1.05 (1.04-1.07) | <0.001 | <0.001 |
| Corn oil (8 g·2000 kcal^-1^·d^-1^) | 1.00 (0.97-1.03) | 0.97 |  | 0.98 (0.94-1.02) | 0.23 | 0.67 |
| Canola oil (8 g·2000 kcal^-1^·d^-1^) | 0.98 (0.95-1.01) | 0.14 |  | 0.97 (0.93-1.00) | 0.078 | 0.96 |
| Olive oil (8 g·2000 kcal^-1^·d^-1^) | 0.98 (0.96-1.01) | 0.26 |  | 0.97 (0.94-1.00) | 0.044 | 0.31 |
|  |  |  |  |  |  |  |
|  | HEI score | | | | |  |
|  | Below median | |  | Above median | | *P* for interaction |
|  | HR (95% CI) | *P* value |  | HR (95% CI) | *P* value |  |
| Butter (14 g·2000 kcal^-1^·d^-1^) | 1.06 (1.05-1.08) | <0.001 |  | 1.11 (1.07-1.14) | <0.001 | 0.27 |
| Margarine (14 g·2000 kcal^-1^·d^-1^) | 1.03 (1.02-1.04) | <0.001 |  | 1.05 (1.04-1.07) | <0.001 | 0.001 |
| Corn oil (8 g·2000 kcal^-1^·d^-1^) | 1.00 (0.97-1.03) | 0.95 |  | 0.98 (0.94-1.02) | 0.25 | 0.87 |
| Canola oil (8 g·2000 kcal^-1^·d^-1^) | 0.96 (0.93-1.00) | 0.040 |  | 0.98 (0.95-1.01) | 0.19 | 0.28 |
| Olive oil (8 g·2000 kcal^-1^·d^-1^) | 0.99 (0.96-1.01) | 0.30 |  | 0.97 (0.94-1.00) | 0.041 | 0.43 |

BMI, body mass index; CI, confidence interval; HEI, Healthy Eating Index; HR, hazard ratio. HRs were adjusted for age, sex, BMI, race, education, marital status, household income, smoking, alcohol, vigorous physical activity, usual activity at work, perceived health condition, history of heart disease, stroke, diabetes, and cancer at baseline, Healthy Eating Index-2015, total energy intake, and consumption of remaining oils where appropriate (butter, margarine, lard, corn oil, canola oil, olive oil and other vegetable oils). P values for interaction were calculated by likelihood-ratio tests comparing Cox proportional-hazards models with and without cross-product terms for each level of baseline stratifying variables.

# Table S10. Multivariable-adjusted HRs (95% CIs) of all-cause and cause-specific mortality from the sensitivity analysis that excluding those with extreme BMIs

|  | Categories of individual oil consumption | | | |  |
| --- | --- | --- | --- | --- | --- |
|  | Non-consumers | T1 | T2 | T3 | *P* trend |
| All-cause |  |  |  |  |  |
| Butter | 1.00 | 0.98 (0.96-1.00) | 1.05 (1.03-1.07) | 1.09 (1.07-1.11) | <0.001 |
| Margarine | 1.00 | 0.98 (0.97-1.00) | 1.02 (1.00-1.04) | 1.06 (1.04-1.09) | <0.001 |
| Corn oil | 1.00 | 0.96 (0.94-0.98) | 0.98 (0.96-1.00) | 0.99 (0.97-1.01) | 0.16 |
| Canola oil | 1.00 | 0.98 (0.95-1.00) | 0.97 (0.95-0.99) | 0.97 (0.95-0.99) | <0.001 |
| Olive oil | 1.00 | 0.97 (0.94-0.99) | 0.97 (0.95-0.99) | 0.96 (0.94-0.98) | <0.001 |
| Cardiovascular disease |  |  |  |  |  |
| Butter | 1.00 | 0.97 (0.93-1.00) | 1.05 (1.01-1.08) | 1.08 (1.05-1.12) | <0.001 |
| Margarine | 1.00 | 1.00 (0.97-1.04) | 1.05 (1.01-1.09) | 1.10 (1.06-1.14) | <0.001 |
| Corn oil | 1.00 | 1.01 (0.96-1.05) | 0.99 (0.95-1.03) | 1.01 (0.97-1.05) | 0.68 |
| Canola oil | 1.00 | 0.99 (0.95-1.04) | 0.98 (0.94-1.02) | 0.97 (0.94-1.01) | 0.11 |
| Olive oil | 1.00 | 0.93 (0.89-0.97) | 0.95 (0.91-0.99) | 0.96 (0.92-0.99) | 0.004 |
| Cancer |  |  |  |  |  |
| Butter | 1.00 | 0.98 (0.95-1.01) | 1.04 (1.01-1.07) | 1.05 (1.02-1.09) | <0.001 |
| Margarine | 1.00 | 0.97 (0.94-1.00) | 0.99 (0.96-1.02) | 1.01 (0.98-1.04) | 0.049 |
| Corn oil | 1.00 | 0.94 (0.90-0.97) | 0.96 (0.93-1.00) | 0.98 (0.94-1.01) | 0.081 |
| Canola oil | 1.00 | 0.98 (0.94-1.02) | 0.97 (0.94-1.01) | 1.00 (0.96-1.03) | 0.58 |
| Olive oil | 1.00 | 1.03 (0.99-1.08) | 1.01 (0.98-1.05) | 1.02 (0.99-1.05) | 0.18 |
| Respiratory disease |  |  |  |  |  |
| Butter | 1.00 | 0.99 (0.92-1.07) | 1.09 (1.02-1.17) | 1.23 (1.15-1.31) | <0.001 |
| Margarine | 1.00 | 0.98 (0.91-1.06) | 1.10 (1.02-1.17) | 1.23 (1.14-1.32) | <0.001 |
| Corn oil | 1.00 | 0.96 (0.87-1.05) | 0.97 (0.90-1.05) | 1.01 (0.94-1.09) | 0.86 |
| Canola oil | 1.00 | 0.96 (0.87-1.05) | 0.94 (0.86-1.02) | 0.95 (0.88-1.02) | 0.087 |
| Olive oil | 1.00 | 0.92 (0.84-1.01) | 0.90 (0.83-0.97) | 0.89 (0.82-0.95) | <0.001 |
| Alzheimer's disease |  |  |  |  |  |
| Butter | 1.00 | 0.94 (0.81-1.10) | 0.92 (0.79-1.07) | 0.93 (0.80-1.09) | 0.36 |
| Margarine | 1.00 | 1.06 (0.91-1.24) | 1.07 (0.93-1.24) | 1.06 (0.91-1.24) | 0.64 |
| Corn oil | 1.00 | 0.96 (0.79-1.16) | 1.04 (0.88-1.23) | 0.85 (0.71-1.02) | 0.12 |
| Canola oil | 1.00 | 1.06 (0.88-1.28) | 1.01 (0.86-1.18) | 0.95 (0.81-1.11) | 0.53 |
| Olive oil | 1.00 | 0.80 (0.66-0.97) | 0.88 (0.75-1.04) | 0.77 (0.65-0.91) | 0.001 |
| Diabetes |  |  |  |  |  |
| Butter | 1.00 | 0.97 (0.85-1.10) | 0.99 (0.88-1.11) | 1.20 (1.07-1.35) | 0.003 |
| Margarine | 1.00 | 1.06 (0.93-1.20) | 1.07 (0.95-1.21) | 1.17 (1.04-1.32) | 0.009 |
| Corn oil | 1.00 | 0.94 (0.80-1.10) | 1.00 (0.88-1.14) | 0.89 (0.78-1.01) | 0.085 |
| Canola oil | 1.00 | 1.09 (0.93-1.27) | 1.04 (0.92-1.19) | 1.00 (0.89-1.12) | 0.92 |
| Olive oil | 1.00 | 0.83 (0.70-0.97) | 0.96 (0.84-1.09) | 0.85 (0.74-0.96) | 0.009 |
| Infections |  |  |  |  |  |
| Butter | 1.00 | 1.02 (0.88-1.18) | 1.05 (0.92-1.20) | 1.10 (0.96-1.26) | 0.17 |
| Margarine | 1.00 | 0.90 (0.78-1.04) | 1.05 (0.92-1.19) | 1.03 (0.89-1.18) | 0.21 |
| Corn oil | 1.00 | 0.96 (0.80-1.15) | 1.29 (1.12-1.49) | 1.00 (0.86-1.15) | 0.53 |
| Canola oil | 1.00 | 0.79 (0.65-0.95) | 0.89 (0.76-1.04) | 0.85 (0.73-0.99) | 0.015 |
| Olive oil | 1.00 | 1.03 (0.86-1.23) | 0.92 (0.79-1.06) | 0.96 (0.83-1.11) | 0.44 |
| Kidney disease |  |  |  |  |  |
| Butter | 1.00 | 0.96 (0.80-1.14) | 1.10 (0.94-1.29) | 1.25 (1.07-1.47) | 0.003 |
| Margarine | 1.00 | 0.99 (0.84-1.18) | 1.05 (0.90-1.23) | 1.20 (1.02-1.41) | 0.007 |
| Corn oil | 1.00 | 0.85 (0.68-1.07) | 0.87 (0.72-1.06) | 1.03 (0.88-1.22) | 0.92 |
| Canola oil | 1.00 | 0.93 (0.75-1.16) | 0.87 (0.72-1.05) | 0.98 (0.83-1.15) | 0.61 |
| Olive oil | 1.00 | 0.93 (0.75-1.16) | 1.01 (0.84-1.21) | 0.99 (0.84-1.16) | 0.87 |
| Chronic liver disease |  |  |  |  |  |
| Butter | 1.00 | 0.88 (0.70-1.11) | 1.20 (0.99-1.45) | 1.36 (1.11-1.66) | <0.001 |
| Margarine | 1.00 | 0.90 (0.73-1.11) | 1.09 (0.90-1.32) | 0.94 (0.75-1.17) | 0.92 |
| Corn oil | 1.00 | 0.89 (0.68-1.17) | 1.03 (0.81-1.30) | 1.08 (0.86-1.35) | 0.53 |
| Canola oil | 1.00 | 0.75 (0.56-1.00) | 0.89 (0.69-1.14) | 0.99 (0.79-1.25) | 0.74 |
| Olive oil | 1.00 | 1.28 (0.99-1.65) | 1.05 (0.84-1.31) | 0.95 (0.77-1.18) | 0.68 |
| Other causes |  |  |  |  |  |
| Butter | 1.00 | 1.00 (0.95-1.04) | 1.04 (1.00-1.09) | 1.09 (1.04-1.14) | <0.001 |
| Margarine | 1.00 | 0.99 (0.95-1.04) | 0.99 (0.95-1.03) | 1.05 (1.00-1.10) | 0.008 |
| Corn oil | 1.00 | 0.96 (0.90-1.01) | 0.96 (0.91-1.01) | 0.99 (0.95-1.04) | 0.49 |
| Canola oil | 1.00 | 0.98 (0.93-1.04) | 0.97 (0.92-1.02) | 0.93 (0.88-0.97) | <0.001 |
| Olive oil | 1.00 | 0.93 (0.88-0.98) | 0.93 (0.89-0.98) | 0.93 (0.89-0.97) | <0.001 |

CI, confidence interval; HR, hazard ratio; T, tertile. HRs were adjusted for age, sex, BMI, race, education, marital status, household income, smoking, alcohol, vigorous physical activity, usual activity at work, perceived health condition, history of heart disease, stroke, diabetes, and cancer at baseline, Healthy Eating Index-2015, total energy intake, and consumption of remaining oils where appropriate (butter, margarine, lard, corn oil, canola oil, olive oil, and other vegetable oils).

# Table S11. Multivariable-adjusted HRs (95% CIs) of all-cause and cause-specific mortality from the sensitivity analysis that further adjusting for a propensity score

|  | Categories of individual oil consumption | | | |  |
| --- | --- | --- | --- | --- | --- |
|  | Non-consumers | T1 | T2 | T3 | *P* trend |
| All-cause |  |  |  |  |  |
| Butter | 1.00 | 0.98 (0.96-1.00) | 1.04 (1.03-1.06) | 1.08 (1.06-1.11) | <0.001 |
| Margarine | 1.00 | 0.99 (0.97-1.01) | 1.03 (1.01-1.05) | 1.07 (1.05-1.09) | <0.001 |
| Corn oil | 1.00 | 0.96 (0.94-0.98) | 0.97 (0.95-0.99) | 0.99 (0.97-1.01) | 0.15 |
| Canola oil | 1.00 | 0.97 (0.95-1.00) | 0.97 (0.95-0.99) | 0.97 (0.95-0.99) | <0.001 |
| Olive oil | 1.00 | 0.96 (0.94-0.98) | 0.97 (0.95-0.99) | 0.96 (0.95-0.98) | <0.001 |
| Cardiovascular disease |  |  |  |  |  |
| Butter | 1.00 | 0.97 (0.93-1.00) | 1.04 (1.01-1.08) | 1.08 (1.04-1.11) | <0.001 |
| Margarine | 1.00 | 1.02 (0.98-1.05) | 1.07 (1.03-1.10) | 1.11 (1.07-1.15) | <0.001 |
| Corn oil | 1.00 | 1.00 (0.96-1.05) | 0.98 (0.95-1.02) | 1.00 (0.96-1.04) | 0.85 |
| Canola oil | 1.00 | 1.00 (0.95-1.04) | 0.98 (0.94-1.02) | 0.97 (0.93-1.00) | 0.038 |
| Olive oil | 1.00 | 0.92 (0.88-0.96) | 0.96 (0.92-0.99) | 0.95 (0.92-0.99) | 0.001 |
| Cancer |  |  |  |  |  |
| Butter | 1.00 | 0.98 (0.95-1.01) | 1.03 (1.00-1.06) | 1.05 (1.02-1.09) | <0.001 |
| Margarine | 1.00 | 0.98 (0.95-1.01) | 1.00 (0.97-1.03) | 1.02 (0.99-1.05) | 0.025 |
| Corn oil | 1.00 | 0.94 (0.90-0.97) | 0.96 (0.93-0.99) | 0.98 (0.95-1.02) | 0.12 |
| Canola oil | 1.00 | 0.97 (0.93-1.01) | 0.97 (0.94-1.00) | 1.00 (0.97-1.03) | 0.68 |
| Olive oil | 1.00 | 1.03 (0.99-1.07) | 1.01 (0.98-1.05) | 1.02 (0.99-1.05) | 0.16 |
| Respiratory disease |  |  |  |  |  |
| Butter | 1.00 | 1.01 (0.94-1.08) | 1.10 (1.04-1.18) | 1.20 (1.13-1.29) | <0.001 |
| Margarine | 1.00 | 0.97 (0.90-1.05) | 1.09 (1.02-1.17) | 1.24 (1.15-1.33) | <0.001 |
| Corn oil | 1.00 | 0.96 (0.88-1.04) | 0.97 (0.90-1.05) | 1.02 (0.95-1.10) | 0.65 |
| Canola oil | 1.00 | 0.95 (0.87-1.04) | 0.93 (0.86-1.01) | 0.95 (0.88-1.02) | 0.074 |
| Olive oil | 1.00 | 0.90 (0.82-0.98) | 0.89 (0.83-0.96) | 0.90 (0.84-0.96) | <0.001 |
| Alzheimer's disease |  |  |  |  |  |
| Butter | 1.00 | 0.94 (0.81-1.09) | 0.94 (0.82-1.09) | 0.97 (0.83-1.13) | 0.65 |
| Margarine | 1.00 | 1.11 (0.95-1.30) | 1.10 (0.94-1.27) | 1.08 (0.92-1.26) | 0.77 |
| Corn oil | 1.00 | 0.97 (0.81-1.17) | 1.00 (0.84-1.18) | 0.84 (0.71-1.01) | 0.069 |
| Canola oil | 1.00 | 1.02 (0.85-1.23) | 1.01 (0.86-1.18) | 0.92 (0.78-1.07) | 0.31 |
| Olive oil | 1.00 | 0.82 (0.68-0.99) | 0.89 (0.76-1.04) | 0.74 (0.63-0.88) | <0.001 |
| Diabetes |  |  |  |  |  |
| Butter | 1.00 | 0.97 (0.86-1.10) | 0.99 (0.89-1.11) | 1.20 (1.07-1.34) | 0.002 |
| Margarine | 1.00 | 1.07 (0.94-1.22) | 1.11 (0.98-1.25) | 1.15 (1.02-1.30) | 0.037 |
| Corn oil | 1.00 | 0.96 (0.83-1.12) | 1.02 (0.90-1.16) | 0.95 (0.84-1.07) | 0.40 |
| Canola oil | 1.00 | 1.08 (0.93-1.25) | 1.08 (0.96-1.22) | 0.98 (0.87-1.09) | 0.89 |
| Olive oil | 1.00 | 0.84 (0.72-0.98) | 0.93 (0.82-1.06) | 0.87 (0.77-0.99) | 0.017 |
| Infections |  |  |  |  |  |
| Butter | 1.00 | 1.01 (0.89-1.16) | 1.06 (0.94-1.20) | 1.10 (0.96-1.26) | 0.15 |
| Margarine | 1.00 | 0.90 (0.78-1.04) | 1.00 (0.88-1.14) | 0.99 (0.86-1.14) | 0.48 |
| Corn oil | 1.00 | 0.98 (0.82-1.16) | 1.30 (1.14-1.49) | 1.00 (0.87-1.15) | 0.47 |
| Canola oil | 1.00 | 0.77 (0.64-0.92) | 0.87 (0.75-1.01) | 0.88 (0.76-1.01) | 0.024 |
| Olive oil | 1.00 | 1.01 (0.85-1.19) | 0.94 (0.81-1.08) | 0.99 (0.86-1.13) | 0.71 |
| Kidney disease |  |  |  |  |  |
| Butter | 1.00 | 0.96 (0.81-1.14) | 1.08 (0.93-1.26) | 1.23 (1.05-1.43) | 0.006 |
| Margarine | 1.00 | 0.94 (0.79-1.11) | 1.02 (0.87-1.20) | 1.13 (0.95-1.33) | 0.023 |
| Corn oil | 1.00 | 0.83 (0.67-1.03) | 0.89 (0.74-1.06) | 1.07 (0.92-1.25) | 0.58 |
| Canola oil | 1.00 | 0.98 (0.79-1.21) | 0.86 (0.72-1.03) | 0.96 (0.82-1.12) | 0.41 |
| Olive oil | 1.00 | 0.89 (0.72-1.10) | 1.00 (0.83-1.19) | 1.01 (0.87-1.18) | 0.90 |
| Chronic liver disease |  |  |  |  |  |
| Butter | 1.00 | 0.91 (0.73-1.13) | 1.19 (0.99-1.43) | 1.31 (1.08-1.60) | 0.002 |
| Margarine | 1.00 | 0.91 (0.73-1.12) | 1.11 (0.92-1.36) | 0.98 (0.78-1.22) | 0.75 |
| Corn oil | 1.00 | 0.91 (0.69-1.19) | 1.06 (0.84-1.33) | 1.08 (0.87-1.35) | 0.45 |
| Canola oil | 1.00 | 0.77 (0.58-1.02) | 0.94 (0.74-1.19) | 1.01 (0.81-1.26) | 0.94 |
| Olive oil | 1.00 | 1.27 (0.99-1.64) | 1.03 (0.83-1.28) | 0.95 (0.77-1.16) | 0.65 |
| Other causes |  |  |  |  |  |
| Butter | 1.00 | 1.00 (0.96-1.05) | 1.05 (1.00-1.09) | 1.08 (1.04-1.13) | <0.001 |
| Margarine | 1.00 | 1.00 (0.96-1.05) | 1.00 (0.96-1.04) | 1.05 (1.01-1.10) | 0.007 |
| Corn oil | 1.00 | 0.96 (0.91-1.02) | 0.95 (0.90-1.00) | 0.99 (0.94-1.04) | 0.37 |
| Canola oil | 1.00 | 0.97 (0.91-1.02) | 0.96 (0.92-1.01) | 0.93 (0.89-0.97) | <0.001 |
| Olive oil | 1.00 | 0.92 (0.87-0.97) | 0.93 (0.89-0.97) | 0.93 (0.89-0.98) | <0.001 |

CI, confidence interval; HR, hazard ratio; T, tertile. HRs were adjusted for age, sex, BMI, race, education, marital status, household income, smoking, alcohol, vigorous physical activity, usual activity at work, perceived health condition, history of heart disease, stroke, diabetes, and cancer at baseline, Healthy Eating Index-2015, total energy intake, consumption of remaining oils where appropriate (butter, margarine, lard, corn oil, canola oil, olive oil, and other vegetable oils), and a propensity score.

# Table S12. Multivariable-adjusted HRs (95% CIs) of all-cause and cause-specific mortality from the sensitivity analysis that further adjusted for history of hypertension and hypercholesteremia

|  | Categories of oil intake | | | |  |
| --- | --- | --- | --- | --- | --- |
|  | Nonconsumers | T1 | T2 | T3 | P trend |
| All-cause |  |  |  |  |  |
| Butter | 1.00 | 0.98 (0.96-1.00) | 1.04 (1.02-1.06) | 1.09 (1.07-1.11) | <0.001 |
| Margarine | 1.00 | 0.99 (0.97-1.01) | 1.03 (1.01-1.05) | 1.07 (1.05-1.09) | <0.001 |
| Corn oil | 1.00 | 0.97 (0.94-0.99) | 0.97 (0.95-1.00) | 0.99 (0.97-1.01) | 0.053 |
| Canola oil | 1.00 | 0.98 (0.96-1.00) | 0.97 (0.95-0.99) | 0.97 (0.95-0.99) | <0.001 |
| Olive oil | 1.00 | 0.96 (0.94-0.99) | 0.97 (0.95-0.99) | 0.97 (0.95-0.98) | <0.001 |
| Cardiovascular disease |  |  |  |  |  |
| Butter | 1.00 | 0.96 (0.93-1.00) | 1.04 (1.01-1.07) | 1.08 (1.04-1.12) | <0.001 |
| Margarine | 1.00 | 1.00 (0.97-1.04) | 1.06 (1.02-1.09) | 1.10 (1.06-1.13) | <0.001 |
| Corn oil | 1.00 | 1.01 (0.96-1.05) | 0.99 (0.95-1.03) | 1.00 (0.96-1.03) | 0.67 |
| Canola oil | 1.00 | 1.00 (0.95-1.04) | 0.98 (0.94-1.02) | 0.97 (0.94-1.00) | 0.057 |
| Olive oil | 1.00 | 0.93 (0.89-0.97) | 0.96 (0.92-0.99) | 0.95 (0.92-0.99) | 0.002 |
| Cancer |  |  |  |  |  |
| Butter | 1.00 | 0.98 (0.95-1.01) | 1.03 (1.00-1.06) | 1.05 (1.02-1.09) | <0.001 |
| Margarine | 1.00 | 0.98 (0.95-1.01) | 1.00 (0.97-1.03) | 1.01 (0.98-1.05) | 0.064 |
| Corn oil | 1.00 | 0.94 (0.91-0.98) | 0.96 (0.93-0.99) | 0.98 (0.94-1.01) | 0.063 |
| Canola oil | 1.00 | 0.98 (0.94-1.02) | 0.97 (0.94-1.01) | 1.00 (0.97-1.04) | 0.85 |
| Olive oil | 1.00 | 1.03 (0.99-1.07) | 1.01 (0.98-1.05) | 1.02 (0.99-1.06) | 0.13 |
| Respiratory disease |  |  |  |  |  |
| Butter | 1.00 | 0.99 (0.92-1.07) | 1.09 (1.03-1.17) | 1.21 (1.14-1.30) | <0.001 |
| Margarine | 1.00 | 0.98 (0.92-1.05) | 1.10 (1.03-1.17) | 1.23 (1.15-1.32) | <0.001 |
| Corn oil | 1.00 | 0.97 (0.89-1.05) | 0.97 (0.90-1.05) | 1.01 (0.94-1.08) | 0.96 |
| Canola oil | 1.00 | 0.98 (0.90-1.07) | 0.94 (0.87-1.01) | 0.95 (0.88-1.02) | 0.072 |
| Olive oil | 1.00 | 0.90 (0.83-0.98) | 0.90 (0.83-0.97) | 0.90 (0.84-0.97) | <0.001 |
| Alzheimer's disease |  |  |  |  |  |
| Butter | 1.00 | 0.94 (0.81-1.09) | 0.93 (0.80-1.07) | 0.94 (0.81-1.10) | 0.42 |
| Margarine | 1.00 | 1.08 (0.94-1.26) | 1.06 (0.92-1.23) | 1.05 (0.91-1.22) | 0.89 |
| Corn oil | 1.00 | 0.98 (0.81-1.18) | 1.01 (0.85-1.19) | 0.83 (0.70-1.00) | 0.058 |
| Canola oil | 1.00 | 1.04 (0.87-1.24) | 1.01 (0.86-1.18) | 0.91 (0.78-1.07) | 0.29 |
| Olive oil | 1.00 | 0.82 (0.68-0.98) | 0.87 (0.75-1.02) | 0.75 (0.64-0.89) | <0.001 |
| Diabetes |  |  |  |  |  |
| Butter | 1.00 | 0.97 (0.86-1.09) | 0.99 (0.89-1.11) | 1.18 (1.05-1.31) | 0.005 |
| Margarine | 1.00 | 1.05 (0.93-1.18) | 1.08 (0.97-1.21) | 1.12 (1.00-1.26) | 0.048 |
| Corn oil | 1.00 | 0.96 (0.83-1.12) | 1.02 (0.90-1.16) | 0.94 (0.83-1.06) | 0.37 |
| Canola oil | 1.00 | 1.07 (0.92-1.24) | 1.08 (0.95-1.22) | 0.98 (0.88-1.10) | 0.92 |
| Olive oil | 1.00 | 0.84 (0.72-0.98) | 0.94 (0.83-1.07) | 0.88 (0.78-0.99) | 0.023 |
| Infections |  |  |  |  |  |
| Butter | 1.00 | 1.02 (0.89-1.16) | 1.06 (0.93-1.20) | 1.10 (0.97-1.26) | 0.12 |
| Margarine | 1.00 | 0.90 (0.79-1.03) | 1.01 (0.90-1.15) | 0.99 (0.87-1.13) | 0.45 |
| Corn oil | 1.00 | 0.98 (0.83-1.17) | 1.28 (1.12-1.47) | 0.99 (0.86-1.14) | 0.54 |
| Canola oil | 1.00 | 0.77 (0.64-0.92) | 0.87 (0.75-1.01) | 0.88 (0.76-1.01) | 0.031 |
| Olive oil | 1.00 | 1.05 (0.89-1.24) | 0.93 (0.81-1.08) | 0.98 (0.85-1.12) | 0.60 |
| Kidney disease |  |  |  |  |  |
| Butter | 1.00 | 0.96 (0.81-1.14) | 1.09 (0.94-1.26) | 1.26 (1.09-1.46) | 0.001 |
| Margarine | 1.00 | 0.97 (0.82-1.14) | 1.05 (0.90-1.22) | 1.17 (1.00-1.36) | 0.010 |
| Corn oil | 1.00 | 0.83 (0.67-1.03) | 0.88 (0.74-1.06) | 1.07 (0.92-1.24) | 0.59 |
| Canola oil | 1.00 | 0.96 (0.78-1.19) | 0.86 (0.71-1.03) | 0.97 (0.83-1.13) | 0.49 |
| Olive oil | 1.00 | 0.90 (0.73-1.11) | 1.00 (0.84-1.19) | 1.01 (0.87-1.18) | 0.92 |
| Chronic liver disease |  |  |  |  |  |
| Butter | 1.00 | 0.90 (0.72-1.12) | 1.18 (0.98-1.42) | 1.32 (1.09-1.61) | 0.001 |
| Margarine | 1.00 | 0.88 (0.72-1.08) | 1.09 (0.91-1.32) | 0.95 (0.77-1.18) | 0.87 |
| Corn oil | 1.00 | 0.89 (0.68-1.16) | 1.05 (0.83-1.32) | 1.09 (0.88-1.35) | 0.43 |
| Canola oil | 1.00 | 0.76 (0.58-1.01) | 0.95 (0.75-1.20) | 1.03 (0.83-1.29) | 0.90 |
| Olive oil | 1.00 | 1.27 (0.99-1.63) | 1.03 (0.83-1.28) | 0.96 (0.78-1.17) | 0.69 |
| Other causes |  |  |  |  |  |
| Butter | 1.00 | 1.00 (0.95-1.04) | 1.04 (1.00-1.09) | 1.08 (1.04-1.13) | <0.001 |
| Margarine | 1.00 | 1.00 (0.96-1.05) | 1.00 (0.96-1.04) | 1.05 (1.01-1.10) | 0.005 |
| Corn oil | 1.00 | 0.97 (0.91-1.02) | 0.95 (0.90-1.00) | 0.98 (0.94-1.03) | 0.27 |
| Canola oil | 1.00 | 0.98 (0.92-1.03) | 0.97 (0.92-1.01) | 0.93 (0.89-0.97) | <0.001 |
| Olive oil | 1.00 | 0.93 (0.88-0.98) | 0.93 (0.89-0.98) | 0.93 (0.89-0.97) | <0.001 |

CI, confidence interval; HR, hazard ratio; T, tertile. HRs were adjusted for age, sex, BMI, race, education, marital status, household income, smoking, alcohol, vigorous physical activity, usual activity at work, perceived health condition, history of heart disease, stroke, diabetes, and cancer at baseline, Healthy Eating Index-2015, total energy intake, consumption of remaining oils where appropriate (butter, margarine, lard, corn oil, canola oil, olive oil and other vegetable oils), history of hypertension (yes or no), and history of hypertension hypercholesteremia (yes or no).

# Table S13. Multivariable-adjusted HRs (95% CIs) of all-cause and cause-specific mortality from the sensitivity analysis that further adjusted for aspirin and multivitamins use

|  | Categories of individual oil consumption | | | |  |
| --- | --- | --- | --- | --- | --- |
|  | Non-consumers | T1 | T2 | T3 | *P* trend |
| All-cause |  |  |  |  |  |
| Butter | 1.00 | 0.98 (0.96-1.00) | 1.05 (1.03-1.07) | 1.09 (1.07-1.11) | <0.001 |
| Margarine | 1.00 | 0.99 (0.97-1.01) | 1.03 (1.01-1.05) | 1.07 (1.05-1.09) | <0.001 |
| Corn oil | 1.00 | 0.97 (0.95-0.99) | 0.98 (0.96-1.00) | 0.99 (0.97-1.01) | 0.24 |
| Canola oil | 1.00 | 0.98 (0.95-1.00) | 0.97 (0.95-0.99) | 0.97 (0.95-0.99) | <0.001 |
| Olive oil | 1.00 | 0.96 (0.94-0.99) | 0.97 (0.95-0.99) | 0.97 (0.95-0.98) | <0.001 |
| Cardiovascular disease |  |  |  |  |  |
| Butter | 1.00 | 0.97 (0.93-1.00) | 1.05 (1.01-1.08) | 1.09 (1.05-1.13) | <0.001 |
| Margarine | 1.00 | 1.01 (0.97-1.04) | 1.06 (1.02-1.09) | 1.10 (1.06-1.14) | <0.001 |
| Corn oil | 1.00 | 1.01 (0.97-1.06) | 1.00 (0.96-1.03) | 1.00 (0.97-1.04) | 0.92 |
| Canola oil | 1.00 | 0.99 (0.95-1.04) | 0.98 (0.94-1.02) | 0.97 (0.94-1.01) | 0.074 |
| Olive oil | 1.00 | 0.93 (0.89-0.97) | 0.96 (0.92-0.99) | 0.95 (0.92-0.99) | 0.002 |
| Cancer |  |  |  |  |  |
| Butter | 1.00 | 0.98 (0.95-1.01) | 1.04 (1.01-1.07) | 1.06 (1.03-1.09) | <0.001 |
| Margarine | 1.00 | 0.98 (0.95-1.01) | 1.00 (0.97-1.03) | 1.02 (0.98-1.05) | 0.051 |
| Corn oil | 1.00 | 0.95 (0.91-0.98) | 0.96 (0.93-1.00) | 0.98 (0.95-1.02) | 0.12 |
| Canola oil | 1.00 | 0.97 (0.94-1.01) | 0.97 (0.94-1.01) | 1.00 (0.97-1.04) | 0.80 |
| Olive oil | 1.00 | 1.03 (0.99-1.07) | 1.01 (0.98-1.05) | 1.02 (0.99-1.06) | 0.14 |
| Respiratory disease |  |  |  |  |  |
| Butter | 1.00 | 1.00 (0.93-1.08) | 1.11 (1.04-1.18) | 1.23 (1.16-1.31) | <0.001 |
| Margarine | 1.00 | 0.98 (0.91-1.05) | 1.09 (1.03-1.17) | 1.23 (1.15-1.32) | <0.001 |
| Corn oil | 1.00 | 0.97 (0.89-1.06) | 0.98 (0.91-1.06) | 1.02 (0.95-1.09) | 0.68 |
| Canola oil | 1.00 | 0.97 (0.89-1.06) | 0.93 (0.86-1.01) | 0.94 (0.88-1.01) | 0.050 |
| Olive oil | 1.00 | 0.90 (0.82-0.98) | 0.89 (0.83-0.96) | 0.90 (0.84-0.96) | <0.001 |
| Alzheimer's disease |  |  |  |  |  |
| Butter | 1.00 | 0.94 (0.81-1.09) | 0.93 (0.80-1.07) | 0.94 (0.81-1.10) | 0.43 |
| Margarine | 1.00 | 1.09 (0.94-1.26) | 1.07 (0.93-1.23) | 1.06 (0.91-1.22) | 0.83 |
| Corn oil | 1.00 | 0.99 (0.82-1.19) | 1.02 (0.86-1.20) | 0.84 (0.71-1.01) | 0.076 |
| Canola oil | 1.00 | 1.04 (0.87-1.24) | 1.01 (0.86-1.18) | 0.92 (0.78-1.07) | 0.30 |
| Olive oil | 1.00 | 0.82 (0.68-0.98) | 0.88 (0.75-1.03) | 0.76 (0.64-0.89) | <0.001 |
| Diabetes |  |  |  |  |  |
| Butter | 1.00 | 0.97 (0.86-1.09) | 0.99 (0.89-1.11) | 1.18 (1.06-1.32) | 0.004 |
| Margarine | 1.00 | 1.04 (0.93-1.18) | 1.08 (0.97-1.21) | 1.12 (1.00-1.26) | 0.044 |
| Corn oil | 1.00 | 0.97 (0.83-1.13) | 1.03 (0.91-1.17) | 0.94 (0.84-1.07) | 0.41 |
| Canola oil | 1.00 | 1.06 (0.92-1.23) | 1.07 (0.95-1.21) | 0.98 (0.88-1.10) | 0.94 |
| Olive oil | 1.00 | 0.84 (0.72-0.98) | 0.94 (0.83-1.06) | 0.87 (0.77-0.99) | 0.019 |
| Infections |  |  |  |  |  |
| Butter | 1.00 | 1.02 (0.89-1.17) | 1.07 (0.95-1.21) | 1.12 (0.98-1.27) | 0.087 |
| Margarine | 1.00 | 0.90 (0.79-1.03) | 1.02 (0.90-1.15) | 1.00 (0.88-1.14) | 0.41 |
| Corn oil | 1.00 | 0.99 (0.84-1.18) | 1.30 (1.14-1.49) | 1.01 (0.88-1.16) | 0.40 |
| Canola oil | 1.00 | 0.77 (0.64-0.92) | 0.87 (0.75-1.01) | 0.88 (0.77-1.02) | 0.032 |
| Olive oil | 1.00 | 1.05 (0.89-1.24) | 0.93 (0.81-1.07) | 0.98 (0.85-1.12) | 0.60 |
| Kidney disease |  |  |  |  |  |
| Butter | 1.00 | 0.96 (0.81-1.14) | 1.10 (0.95-1.28) | 1.27 (1.09-1.47) | 0.001 |
| Margarine | 1.00 | 0.97 (0.83-1.14) | 1.05 (0.91-1.22) | 1.17 (1.01-1.37) | 0.008 |
| Corn oil | 1.00 | 0.84 (0.67-1.04) | 0.90 (0.75-1.08) | 1.08 (0.92-1.25) | 0.50 |
| Canola oil | 1.00 | 0.96 (0.78-1.19) | 0.86 (0.72-1.03) | 0.98 (0.84-1.14) | 0.55 |
| Olive oil | 1.00 | 0.90 (0.73-1.12) | 1.00 (0.84-1.19) | 1.01 (0.87-1.18) | 0.92 |
| Chronic liver disease |  |  |  |  |  |
| Butter | 1.00 | 0.90 (0.72-1.13) | 1.19 (0.99-1.44) | 1.35 (1.11-1.63) | <0.001 |
| Margarine | 1.00 | 0.88 (0.72-1.08) | 1.09 (0.90-1.31) | 0.95 (0.77-1.18) | 0.86 |
| Corn oil | 1.00 | 0.90 (0.69-1.17) | 1.06 (0.84-1.33) | 1.10 (0.88-1.36) | 0.37 |
| Canola oil | 1.00 | 0.76 (0.57-1.00) | 0.93 (0.74-1.18) | 1.02 (0.82-1.27) | 0.99 |
| Olive oil | 1.00 | 1.26 (0.98-1.62) | 1.02 (0.82-1.26) | 0.95 (0.77-1.17) | 0.63 |
| Other causes |  |  |  |  |  |
| Butter | 1.00 | 1.00 (0.96-1.04) | 1.05 (1.01-1.09) | 1.09 (1.04-1.14) | <0.001 |
| Margarine | 1.00 | 1.00 (0.96-1.05) | 1.00 (0.96-1.04) | 1.06 (1.01-1.10) | 0.003 |
| Corn oil | 1.00 | 0.97 (0.92-1.03) | 0.96 (0.91-1.01) | 0.99 (0.95-1.04) | 0.44 |
| Canola oil | 1.00 | 0.97 (0.92-1.03) | 0.96 (0.92-1.01) | 0.93 (0.89-0.97) | <0.001 |
| Olive oil | 1.00 | 0.93 (0.88-0.98) | 0.93 (0.89-0.98) | 0.93 (0.89-0.97) | <0.001 |

CI, confidence interval; HR, hazard ratio; T, tertile. HRs were adjusted for age, sex, BMI, race, education, marital status, household income, smoking, alcohol, vigorous physical activity, usual activity at work, perceived health condition, history of heart disease, stroke, diabetes, and cancer at baseline, Healthy Eating Index-2015, total energy intake, consumption of remaining oils where appropriate (butter, margarine, lard, corn oil, canola oil, olive oil and other vegetable oils), aspirin use (yes or no), and multivitamins use (yes or no).

# Table S14. Multivariable-adjusted HRs (95% CIs) of all-cause and cause-specific mortality from the sensitivity analysis that further adjusted for the use of cholesterol-lowering medications (n=293,918)

|  | Categories of oil intake | | | |  |
| --- | --- | --- | --- | --- | --- |
|  | Nonconsumers | T1 | T2 | T3 | P trend |
| All-cause |  |  |  |  |  |
| Butter | 1.00 | 0.98 (0.94-1.01) | 1.01 (0.98-1.04) | 1.04 (1.01-1.08) | 0.006 |
| Margarine | 1.00 | 0.98 (0.95-1.01) | 1.02 (0.98-1.05) | 1.03 (1.00-1.07) | 0.006 |
| Corn oil | 1.00 | 0.98 (0.94-1.02) | 0.99 (0.96-1.03) | 0.98 (0.95-1.01) | 0.20 |
| Canola oil | 1.00 | 0.97 (0.93-1.01) | 0.96 (0.92-1.00) | 0.97 (0.93-1.01) | 0.034 |
| Olive oil | 1.00 | 0.98 (0.94-1.02) | 0.96 (0.93-1.00) | 0.96 (0.93-0.99) | 0.003 |
| Cardiovascular disease |  |  |  |  |  |
| Butter | 1.00 | 0.99 (0.93-1.05) | 1.00 (0.94-1.06) | 1.02 (0.96-1.09) | 0.45 |
| Margarine | 1.00 | 0.97 (0.91-1.03) | 1.05 (0.99-1.11) | 1.06 (1.00-1.13) | 0.006 |
| Corn oil | 1.00 | 1.01 (0.94-1.09) | 1.01 (0.94-1.08) | 0.99 (0.93-1.05) | 0.72 |
| Canola oil | 1.00 | 1.00 (0.93-1.09) | 0.98 (0.92-1.05) | 0.98 (0.92-1.05) | 0.59 |
| Olive oil | 1.00 | 0.94 (0.87-1.01) | 0.96 (0.90-1.03) | 0.95 (0.89-1.01) | 0.073 |
| Cancer |  |  |  |  |  |
| Butter | 1.00 | 0.97 (0.92-1.03) | 0.97 (0.92-1.02) | 1.01 (0.95-1.06) | 0.84 |
| Margarine | 1.00 | 0.98 (0.93-1.04) | 1.00 (0.94-1.05) | 0.97 (0.91-1.02) | 0.32 |
| Corn oil | 1.00 | 0.98 (0.91-1.05) | 0.95 (0.89-1.02) | 0.95 (0.89-1.02) | 0.078 |
| Canola oil | 1.00 | 0.94 (0.88-1.01) | 1.01 (0.95-1.07) | 0.99 (0.94-1.05) | 0.84 |
| Olive oil | 1.00 | 1.05 (0.98-1.13) | 0.99 (0.93-1.05) | 1.02 (0.97-1.08) | 0.53 |
| Respiratory disease |  |  |  |  |  |
| Butter | 1.00 | 0.91 (0.80-1.03) | 1.00 (0.90-1.12) | 1.13 (1.01-1.26) | 0.015 |
| Margarine | 1.00 | 0.95 (0.85-1.08) | 0.99 (0.89-1.11) | 1.09 (0.97-1.22) | 0.030 |
| Corn oil | 1.00 | 0.85 (0.73-0.99) | 0.92 (0.81-1.05) | 1.01 (0.90-1.14) | 0.94 |
| Canola oil | 1.00 | 1.05 (0.90-1.22) | 0.94 (0.83-1.07) | 1.03 (0.91-1.15) | 0.82 |
| Olive oil | 1.00 | 0.93 (0.81-1.08) | 1.01 (0.90-1.15) | 0.87 (0.77-0.98) | 0.031 |
| Alzheimer's disease |  |  |  |  |  |
| Butter | 1.00 | 0.94 (0.72-1.23) | 0.97 (0.75-1.25) | 0.97 (0.74-1.28) | 0.86 |
| Margarine | 1.00 | 1.06 (0.82-1.38) | 1.09 (0.84-1.40) | 1.05 (0.81-1.38) | 0.85 |
| Corn oil | 1.00 | 0.94 (0.66-1.33) | 1.26 (0.96-1.66) | 0.86 (0.63-1.17) | 0.58 |
| Canola oil | 1.00 | 0.81 (0.58-1.14) | 0.92 (0.70-1.22) | 0.88 (0.67-1.16) | 0.32 |
| Olive oil | 1.00 | 0.86 (0.62-1.20) | 0.79 (0.60-1.05) | 0.74 (0.55-0.98) | 0.019 |
| Diabetes |  |  |  |  |  |
| Butter | 1.00 | 0.94 (0.76-1.17) | 0.95 (0.77-1.16) | 1.06 (0.86-1.30) | 0.62 |
| Margarine | 1.00 | 1.19 (0.96-1.47) | 1.07 (0.87-1.31) | 1.20 (0.97-1.47) | 0.24 |
| Corn oil | 1.00 | 0.93 (0.71-1.21) | 0.94 (0.74-1.18) | 0.90 (0.72-1.12) | 0.29 |
| Canola oil | 1.00 | 1.25 (0.98-1.61) | 1.13 (0.91-1.41) | 1.01 (0.83-1.24) | 0.74 |
| Olive oil | 1.00 | 0.80 (0.62-1.05) | 0.92 (0.73-1.15) | 0.88 (0.71-1.09) | 0.18 |
| Infections |  |  |  |  |  |
| Butter | 1.00 | 0.99 (0.78-1.25) | 0.99 (0.80-1.24) | 1.17 (0.93-1.46) | 0.19 |
| Margarine | 1.00 | 0.94 (0.75-1.19) | 1.04 (0.84-1.29) | 0.91 (0.72-1.15) | 0.48 |
| Corn oil | 1.00 | 1.13 (0.85-1.51) | 1.09 (0.84-1.40) | 1.10 (0.87-1.40) | 0.37 |
| Canola oil | 1.00 | 0.85 (0.63-1.14) | 0.85 (0.65-1.10) | 0.94 (0.74-1.19) | 0.42 |
| Olive oil | 1.00 | 0.95 (0.71-1.26) | 1.05 (0.83-1.34) | 0.87 (0.69-1.11) | 0.32 |
| Kidney disease |  |  |  |  |  |
| Butter | 1.00 | 0.91 (0.68-1.21) | 1.20 (0.93-1.53) | 1.36 (1.06-1.75) | 0.007 |
| Margarine | 1.00 | 1.06 (0.81-1.38) | 1.08 (0.84-1.39) | 1.36 (1.05-1.76) | 0.008 |
| Corn oil | 1.00 | 0.62 (0.41-0.94) | 1.00 (0.75-1.33) | 1.00 (0.77-1.30) | 1.00 |
| Canola oil | 1.00 | 0.78 (0.53-1.15) | 0.75 (0.56-1.02) | 1.00 (0.78-1.28) | 0.71 |
| Olive oil | 1.00 | 0.93 (0.64-1.35) | 1.19 (0.92-1.55) | 1.02 (0.79-1.30) | 0.73 |
| Chronic liver disease |  |  |  |  |  |
| Butter | 1.00 | 0.85 (0.56-1.29) | 1.09 (0.76-1.56) | 1.39 (0.97-1.99) | 0.050 |
| Margarine | 1.00 | 1.09 (0.75-1.60) | 1.12 (0.78-1.61) | 1.18 (0.79-1.75) | 0.47 |
| Corn oil | 1.00 | 0.82 (0.49-1.36) | 0.84 (0.53-1.32) | 0.82 (0.53-1.28) | 0.31 |
| Canola oil | 1.00 | 1.00 (0.61-1.64) | 1.06 (0.70-1.61) | 1.04 (0.70-1.55) | 0.80 |
| Olive oil | 1.00 | 0.99 (0.62-1.60) | 0.97 (0.65-1.45) | 0.90 (0.61-1.33) | 0.60 |
| Other causes |  |  |  |  |  |
| Butter | 1.00 | 1.01 (0.93-1.09) | 1.08 (1.01-1.16) | 1.06 (0.98-1.14) | 0.10 |
| Margarine | 1.00 | 0.98 (0.91-1.06) | 1.00 (0.93-1.07) | 1.05 (0.97-1.13) | 0.090 |
| Corn oil | 1.00 | 0.99 (0.90-1.09) | 0.92 (0.84-1.01) | 0.97 (0.89-1.05) | 0.25 |
| Canola oil | 1.00 | 0.98 (0.89-1.07) | 0.99 (0.91-1.07) | 0.93 (0.86-1.01) | 0.076 |
| Olive oil | 1.00 | 0.97 (0.88-1.06) | 0.89 (0.82-0.97) | 0.92 (0.86-1.00) | 0.013 |

CI, confidence interval; HR, hazard ratio; T, tertile. HRs were adjusted for age, sex, BMI, race, education, marital status, household income, smoking, alcohol, vigorous physical activity, usual activity at work, perceived health condition, history of heart disease, stroke, diabetes, and cancer at baseline, Healthy Eating Index-2015, total energy intake, consumption of remaining oils where appropriate (butter, margarine, lard, corn oil, canola oil, olive oil and other vegetable oils), use of cholesterol-lowering medications (yes or no).

# Table S15. Multivariable-adjusted HRs (95% CIs) of all-cause and cause-specific mortality from the sensitivity analysis that excluding those with cardiovascular disease, cancer, or diabetes at baseline

|  | Categories of individual oil consumption | | | |  |
| --- | --- | --- | --- | --- | --- |
|  | Non-consumers | T1 | T2 | T3 | *P* trend |
| All-cause |  |  |  |  |  |
| Butter | 1.00 | 0.98 (0.95-1.00) | 1.02 (1.00-1.05) | 1.07 (1.05-1.10) | <0.001 |
| Margarine | 1.00 | 0.99 (0.96-1.01) | 1.02 (0.99-1.04) | 1.05 (1.02-1.08) | <0.001 |
| Corn oil | 1.00 | 0.96 (0.93-0.99) | 0.96 (0.94-0.99) | 0.98 (0.95-1.01) | 0.045 |
| Canola oil | 1.00 | 0.96 (0.93-1.00) | 0.97 (0.95-1.00) | 0.97 (0.94-1.00) | 0.007 |
| Olive oil | 1.00 | 0.96 (0.94-1.00) | 0.97 (0.94-0.99) | 0.96 (0.93-0.98) | <0.001 |
| Cardiovascular disease |  |  |  |  |  |
| Butter | 1.00 | 0.95 (0.90-1.00) | 1.01 (0.96-1.06) | 1.06 (1.01-1.12) | 0.006 |
| Margarine | 1.00 | 1.00 (0.95-1.05) | 1.04 (0.99-1.09) | 1.06 (1.01-1.12) | 0.005 |
| Corn oil | 1.00 | 0.98 (0.92-1.04) | 0.96 (0.91-1.02) | 0.98 (0.93-1.04) | 0.34 |
| Canola oil | 1.00 | 0.97 (0.91-1.04) | 0.97 (0.92-1.03) | 0.99 (0.94-1.04) | 0.48 |
| Olive oil | 1.00 | 0.92 (0.86-0.98) | 0.95 (0.90-1.00) | 0.93 (0.88-0.98) | 0.002 |
| Cancer |  |  |  |  |  |
| Butter | 1.00 | 0.97 (0.93-1.01) | 1.01 (0.97-1.05) | 1.03 (0.99-1.07) | 0.084 |
| Margarine | 1.00 | 0.98 (0.94-1.02) | 0.99 (0.96-1.03) | 1.00 (0.96-1.04) | 0.56 |
| Corn oil | 1.00 | 0.96 (0.91-1.01) | 0.97 (0.93-1.02) | 0.96 (0.92-1.01) | 0.049 |
| Canola oil | 1.00 | 0.96 (0.91-1.01) | 0.96 (0.92-1.01) | 1.02 (0.98-1.07) | 0.58 |
| Olive oil | 1.00 | 1.05 (1.00-1.10) | 1.01 (0.97-1.05) | 1.03 (0.99-1.07) | 0.14 |
| Respiratory disease |  |  |  |  |  |
| Butter | 1.00 | 1.01 (0.92-1.11) | 1.11 (1.02-1.20) | 1.25 (1.15-1.35) | <0.001 |
| Margarine | 1.00 | 0.99 (0.90-1.08) | 1.06 (0.98-1.15) | 1.23 (1.13-1.34) | <0.001 |
| Corn oil | 1.00 | 0.94 (0.84-1.05) | 0.97 (0.88-1.07) | 1.04 (0.95-1.13) | 0.52 |
| Canola oil | 1.00 | 0.98 (0.88-1.10) | 1.03 (0.93-1.13) | 0.89 (0.81-0.98) | 0.022 |
| Olive oil | 1.00 | 0.88 (0.78-0.98) | 0.87 (0.79-0.96) | 0.86 (0.78-0.94) | <0.001 |
| Alzheimer's disease |  |  |  |  |  |
| Butter | 1.00 | 0.91 (0.76-1.09) | 0.89 (0.74-1.05) | 0.91 (0.76-1.09) | 0.30 |
| Margarine | 1.00 | 1.04 (0.87-1.24) | 1.05 (0.89-1.25) | 1.02 (0.86-1.22) | 0.95 |
| Corn oil | 1.00 | 0.98 (0.78-1.23) | 0.96 (0.79-1.17) | 0.83 (0.67-1.02) | 0.079 |
| Canola oil | 1.00 | 0.99 (0.79-1.24) | 1.08 (0.90-1.29) | 0.86 (0.71-1.04) | 0.19 |
| Olive oil | 1.00 | 0.74 (0.59-0.93) | 0.85 (0.70-1.02) | 0.69 (0.57-0.85) | <0.001 |
| Diabetes |  |  |  |  |  |
| Butter | 1.00 | 0.95 (0.72-1.24) | 1.15 (0.90-1.46) | 1.42 (1.11-1.81) | 0.003 |
| Margarine | 1.00 | 1.41 (1.08-1.83) | 1.28 (0.99-1.65) | 1.36 (1.04-1.79) | 0.23 |
| Corn oil | 1.00 | 0.97 (0.70-1.33) | 1.03 (0.78-1.36) | 0.98 (0.75-1.29) | 0.93 |
| Canola oil | 1.00 | 1.13 (0.85-1.49) | 1.23 (0.95-1.60) | 1.25 (0.97-1.60) | 0.065 |
| Olive oil | 1.00 | 1.18 (0.86-1.60) | 0.96 (0.72-1.27) | 0.83 (0.63-1.11) | 0.20 |
| Infections |  |  |  |  |  |
| Butter | 1.00 | 0.91 (0.76-1.10) | 1.11 (0.94-1.31) | 1.06 (0.89-1.26) | 0.32 |
| Margarine | 1.00 | 1.00 (0.83-1.19) | 1.08 (0.91-1.28) | 1.04 (0.87-1.25) | 0.58 |
| Corn oil | 1.00 | 0.90 (0.71-1.14) | 1.35 (1.13-1.62) | 0.95 (0.79-1.15) | 0.90 |
| Canola oil | 1.00 | 0.81 (0.63-1.02) | 0.88 (0.72-1.07) | 0.94 (0.78-1.14) | 0.36 |
| Olive oil | 1.00 | 1.13 (0.91-1.41) | 0.90 (0.74-1.09) | 0.99 (0.82-1.18) | 0.73 |
| Kidney disease |  |  |  |  |  |
| Butter | 1.00 | 1.09 (0.85-1.39) | 1.12 (0.89-1.41) | 1.16 (0.91-1.47) | 0.24 |
| Margarine | 1.00 | 0.97 (0.76-1.25) | 0.98 (0.77-1.24) | 1.19 (0.93-1.52) | 0.072 |
| Corn oil | 1.00 | 0.91 (0.66-1.26) | 0.87 (0.66-1.15) | 0.89 (0.69-1.16) | 0.31 |
| Canola oil | 1.00 | 0.92 (0.66-1.27) | 0.87 (0.65-1.15) | 0.99 (0.77-1.27) | 0.76 |
| Olive oil | 1.00 | 0.88 (0.64-1.22) | 1.19 (0.93-1.52) | 0.89 (0.69-1.15) | 0.55 |
| Chronic liver disease |  |  |  |  |  |
| Butter | 1.00 | 0.98 (0.74-1.29) | 1.10 (0.87-1.40) | 1.51 (1.18-1.93) | <0.001 |
| Margarine | 1.00 | 0.92 (0.71-1.20) | 1.36 (1.07-1.72) | 1.05 (0.79-1.38) | 0.42 |
| Corn oil | 1.00 | 1.07 (0.77-1.49) | 1.12 (0.84-1.49) | 1.18 (0.90-1.55) | 0.19 |
| Canola oil | 1.00 | 0.80 (0.56-1.13) | 1.09 (0.81-1.46) | 0.98 (0.73-1.32) | 0.97 |
| Olive oil | 1.00 | 1.17 (0.85-1.60) | 0.98 (0.74-1.29) | 1.02 (0.79-1.33) | 0.89 |
| Other causes |  |  |  |  |  |
| Butter | 1.00 | 1.01 (0.96-1.07) | 1.03 (0.97-1.08) | 1.09 (1.03-1.15) | 0.003 |
| Margarine | 1.00 | 0.99 (0.93-1.04) | 0.98 (0.93-1.04) | 1.04 (0.99-1.10) | 0.034 |
| Corn oil | 1.00 | 0.95 (0.88-1.02) | 0.91 (0.85-0.97) | 0.99 (0.93-1.05) | 0.34 |
| Canola oil | 1.00 | 0.98 (0.92-1.06) | 0.97 (0.91-1.03) | 0.89 (0.83-0.94) | <0.001 |
| Olive oil | 1.00 | 0.90 (0.84-0.97) | 0.95 (0.90-1.01) | 0.92 (0.87-0.97) | 0.001 |

CI, confidence interval; HR, hazard ratio; T, tertile. HRs were adjusted for age, sex, BMI, race, education, marital status, household income, smoking, alcohol, vigorous physical activity, usual activity at work, perceived health condition, history of heart disease, stroke, diabetes, and cancer at baseline, Healthy Eating Index-2015, total energy intake, and consumption of remaining oils where appropriate (butter, margarine, lard, corn oil, canola oil, olive oil, and other vegetable oils).

# Table S16. Multivariable-adjusted HRs (95% CIs) of all-cause and cause-specific mortality from the sensitivity analysis that excluding the first 4 years of follow-up

|  | Categories of individual oil consumption | | | |  |
| --- | --- | --- | --- | --- | --- |
|  | Non-consumers | T1 | T2 | T3 | *P* trend |
| All-cause |  |  |  |  |  |
| Butter | 1.00 | 0.98 (0.96-1.00) | 1.04 (1.02-1.06) | 1.08 (1.06-1.10) | <0.001 |
| Margarine | 1.00 | 0.99 (0.97-1.01) | 1.02 (1.01-1.04) | 1.06 (1.04-1.08) | <0.001 |
| Corn oil | 1.00 | 0.96 (0.94-0.99) | 0.97 (0.95-1.00) | 0.98 (0.96-1.01) | 0.045 |
| Canola oil | 1.00 | 0.98 (0.96-1.01) | 0.97 (0.94-0.99) | 0.97 (0.95-0.99) | <0.001 |
| Olive oil | 1.00 | 0.96 (0.94-0.98) | 0.96 (0.94-0.98) | 0.96 (0.94-0.98) | <0.001 |
| Cardiovascular disease |  |  |  |  |  |
| Butter | 1.00 | 0.96 (0.93-1.00) | 1.05 (1.01-1.08) | 1.07 (1.04-1.11) | <0.001 |
| Margarine | 1.00 | 1.01 (0.97-1.04) | 1.05 (1.02-1.09) | 1.09 (1.05-1.13) | <0.001 |
| Corn oil | 1.00 | 1.00 (0.96-1.05) | 0.99 (0.95-1.03) | 0.98 (0.95-1.02) | 0.40 |
| Canola oil | 1.00 | 1.02 (0.97-1.06) | 0.98 (0.95-1.02) | 0.97 (0.93-1.00) | 0.069 |
| Olive oil | 1.00 | 0.92 (0.88-0.96) | 0.95 (0.92-0.99) | 0.96 (0.93-1.00) | 0.012 |
| Cancer |  |  |  |  |  |
| Butter | 1.00 | 0.97 (0.94-1.01) | 1.03 (1.00-1.06) | 1.04 (1.00-1.07) | 0.007 |
| Margarine | 1.00 | 0.97 (0.94-1.01) | 1.00 (0.97-1.03) | 1.00 (0.97-1.04) | 0.26 |
| Corn oil | 1.00 | 0.94 (0.90-0.98) | 0.96 (0.92-1.00) | 0.98 (0.94-1.01) | 0.085 |
| Canola oil | 1.00 | 0.97 (0.93-1.01) | 0.97 (0.93-1.00) | 1.01 (0.97-1.04) | 0.95 |
| Olive oil | 1.00 | 1.03 (0.99-1.08) | 1.01 (0.98-1.05) | 1.02 (0.99-1.05) | 0.27 |
| Respiratory disease |  |  |  |  |  |
| Butter | 1.00 | 1.00 (0.93-1.08) | 1.09 (1.02-1.16) | 1.21 (1.13-1.30) | <0.001 |
| Margarine | 1.00 | 0.98 (0.92-1.06) | 1.08 (1.01-1.16) | 1.21 (1.12-1.30) | <0.001 |
| Corn oil | 1.00 | 0.96 (0.87-1.05) | 0.98 (0.90-1.06) | 1.01 (0.94-1.09) | 0.83 |
| Canola oil | 1.00 | 0.98 (0.89-1.07) | 0.94 (0.87-1.02) | 0.96 (0.89-1.03) | 0.14 |
| Olive oil | 1.00 | 0.89 (0.82-0.98) | 0.88 (0.81-0.95) | 0.89 (0.83-0.96) | <0.001 |
| Alzheimer's disease |  |  |  |  |  |
| Butter | 1.00 | 0.94 (0.81-1.09) | 0.93 (0.81-1.08) | 0.94 (0.81-1.09) | 0.41 |
| Margarine | 1.00 | 1.08 (0.94-1.26) | 1.08 (0.93-1.24) | 1.05 (0.91-1.22) | 0.83 |
| Corn oil | 1.00 | 0.97 (0.81-1.17) | 1.02 (0.86-1.20) | 0.84 (0.70-1.00) | 0.072 |
| Canola oil | 1.00 | 1.03 (0.86-1.24) | 0.99 (0.84-1.15) | 0.92 (0.78-1.07) | 0.27 |
| Olive oil | 1.00 | 0.81 (0.67-0.97) | 0.88 (0.75-1.03) | 0.75 (0.64-0.89) | <0.001 |
| Diabetes |  |  |  |  |  |
| Butter | 1.00 | 0.99 (0.87-1.12) | 1.02 (0.91-1.15) | 1.19 (1.06-1.33) | 0.003 |
| Margarine | 1.00 | 1.05 (0.92-1.19) | 1.08 (0.96-1.21) | 1.11 (0.99-1.25) | 0.064 |
| Corn oil | 1.00 | 0.98 (0.84-1.15) | 1.03 (0.91-1.18) | 0.93 (0.82-1.05) | 0.31 |
| Canola oil | 1.00 | 1.10 (0.94-1.28) | 1.07 (0.94-1.21) | 1.02 (0.90-1.14) | 0.66 |
| Olive oil | 1.00 | 0.82 (0.70-0.96) | 0.96 (0.84-1.09) | 0.89 (0.78-1.01) | 0.049 |
| Infections |  |  |  |  |  |
| Butter | 1.00 | 1.04 (0.90-1.20) | 1.08 (0.95-1.23) | 1.11 (0.97-1.27) | 0.13 |
| Margarine | 1.00 | 0.90 (0.79-1.04) | 0.99 (0.87-1.13) | 0.99 (0.86-1.14) | 0.51 |
| Corn oil | 1.00 | 0.97 (0.81-1.16) | 1.28 (1.11-1.47) | 1.00 (0.86-1.16) | 0.50 |
| Canola oil | 1.00 | 0.79 (0.66-0.95) | 0.87 (0.74-1.02) | 0.89 (0.77-1.03) | 0.047 |
| Olive oil | 1.00 | 1.03 (0.87-1.23) | 0.93 (0.81-1.08) | 0.94 (0.82-1.09) | 0.35 |
| Kidney disease |  |  |  |  |  |
| Butter | 1.00 | 0.99 (0.83-1.17) | 1.11 (0.95-1.29) | 1.26 (1.08-1.47) | 0.002 |
| Margarine | 1.00 | 0.95 (0.81-1.12) | 1.04 (0.89-1.21) | 1.18 (1.01-1.38) | 0.005 |
| Corn oil | 1.00 | 0.79 (0.63-0.99) | 0.90 (0.74-1.08) | 1.07 (0.91-1.25) | 0.58 |
| Canola oil | 1.00 | 0.94 (0.75-1.17) | 0.87 (0.72-1.05) | 1.00 (0.85-1.16) | 0.72 |
| Olive oil | 1.00 | 0.93 (0.75-1.16) | 0.98 (0.82-1.17) | 0.98 (0.84-1.15) | 0.81 |
| Chronic liver disease |  |  |  |  |  |
| Butter | 1.00 | 0.92 (0.73-1.16) | 1.13 (0.93-1.38) | 1.31 (1.06-1.61) | 0.006 |
| Margarine | 1.00 | 0.87 (0.70-1.08) | 1.05 (0.86-1.29) | 0.90 (0.72-1.13) | 0.73 |
| Corn oil | 1.00 | 0.90 (0.68-1.20) | 1.02 (0.79-1.31) | 1.13 (0.89-1.42) | 0.33 |
| Canola oil | 1.00 | 0.80 (0.60-1.08) | 0.92 (0.71-1.18) | 1.02 (0.81-1.29) | 0.99 |
| Olive oil | 1.00 | 1.18 (0.90-1.54) | 1.06 (0.84-1.33) | 0.98 (0.79-1.22) | 0.93 |
| Other causes |  |  |  |  |  |
| Butter | 1.00 | 1.00 (0.95-1.04) | 1.03 (0.99-1.08) | 1.08 (1.03-1.13) | <0.001 |
| Margarine | 1.00 | 1.01 (0.96-1.06) | 1.01 (0.96-1.05) | 1.06 (1.01-1.11) | 0.010 |
| Corn oil | 1.00 | 0.97 (0.91-1.03) | 0.93 (0.89-0.99) | 0.99 (0.94-1.04) | 0.35 |
| Canola oil | 1.00 | 0.97 (0.92-1.03) | 0.96 (0.91-1.01) | 0.92 (0.88-0.97) | <0.001 |
| Olive oil | 1.00 | 0.92 (0.87-0.98) | 0.93 (0.88-0.98) | 0.92 (0.87-0.96) | <0.001 |

CI, confidence interval; HR, hazard ratio; T, tertile. HRs were adjusted for age, sex, BMI, race, education, marital status, household income, smoking, alcohol, vigorous physical activity, usual activity at work, perceived health condition, history of heart disease, stroke, diabetes, and cancer at baseline, Healthy Eating Index-2015, total energy intake, and consumption of remaining oils where appropriate (butter, margarine, lard, corn oil, canola oil, olive oil, and other vegetable oils).

# Table S17. Multivariable-adjusted HRs (95% CIs) of all-cause and cause-specific mortality from the sensitivity analysis that followed up for 8 years

|  | Categories of individual oil consumption | | | |  |
| --- | --- | --- | --- | --- | --- |
|  | Non-consumers | T1 | T2 | T3 | *P* trend |
| All-cause |  |  |  |  |  |
| Butter | 1.00 | 0.98 (0.95-1.01) | 1.07 (1.04-1.10) | 1.12 (1.09-1.15) | <0.001 |
| Margarine | 1.00 | 0.99 (0.96-1.02) | 1.03 (1.00-1.06) | 1.09 (1.06-1.12) | <0.001 |
| Corn oil | 1.00 | 0.96 (0.92-0.99) | 0.98 (0.95-1.02) | 1.00 (0.97-1.03) | 0.63 |
| Canola oil | 1.00 | 0.98 (0.94-1.02) | 0.95 (0.92-0.99) | 0.96 (0.93-0.99) | <0.001 |
| Olive oil | 1.00 | 0.97 (0.94-1.01) | 0.96 (0.93-0.99) | 0.97 (0.95-1.00) | 0.036 |
| Cardiovascular disease |  |  |  |  |  |
| Butter | 1.00 | 0.95 (0.90-1.01) | 1.06 (1.01-1.12) | 1.12 (1.07-1.19) | <0.001 |
| Margarine | 1.00 | 1.01 (0.96-1.07) | 1.06 (1.01-1.12) | 1.13 (1.07-1.19) | <0.001 |
| Corn oil | 1.00 | 1.01 (0.94-1.08) | 0.97 (0.92-1.04) | 1.03 (0.97-1.09) | 0.45 |
| Canola oil | 1.00 | 1.04 (0.97-1.11) | 0.94 (0.88-1.00) | 0.96 (0.90-1.01) | 0.052 |
| Olive oil | 1.00 | 0.90 (0.84-0.96) | 0.95 (0.90-1.01) | 0.93 (0.88-0.99) | 0.007 |
| Cancer |  |  |  |  |  |
| Butter | 1.00 | 0.96 (0.92-1.01) | 1.05 (1.01-1.10) | 1.08 (1.03-1.13) | <0.001 |
| Margarine | 1.00 | 0.97 (0.92-1.01) | 0.98 (0.94-1.03) | 1.03 (0.99-1.08) | 0.014 |
| Corn oil | 1.00 | 0.93 (0.87-0.98) | 0.97 (0.92-1.02) | 0.97 (0.92-1.02) | 0.17 |
| Canola oil | 1.00 | 0.97 (0.92-1.04) | 0.98 (0.93-1.04) | 1.00 (0.95-1.05) | 0.92 |
| Olive oil | 1.00 | 1.02 (0.96-1.08) | 1.00 (0.95-1.05) | 1.02 (0.97-1.07) | 0.46 |
| Respiratory disease |  |  |  |  |  |
| Butter | 1.00 | 1.02 (0.90-1.16) | 1.19 (1.06-1.33) | 1.23 (1.10-1.37) | <0.001 |
| Margarine | 1.00 | 1.00 (0.88-1.13) | 1.16 (1.04-1.30) | 1.31 (1.16-1.47) | <0.001 |
| Corn oil | 1.00 | 1.07 (0.93-1.24) | 1.04 (0.92-1.19) | 1.06 (0.95-1.19) | 0.26 |
| Canola oil | 1.00 | 0.89 (0.76-1.04) | 0.84 (0.73-0.97) | 0.88 (0.77-0.99) | 0.012 |
| Olive oil | 1.00 | 0.94 (0.81-1.09) | 0.88 (0.77-1.01) | 0.91 (0.81-1.03) | 0.079 |
| Alzheimer's disease |  |  |  |  |  |
| Butter | 1.00 | 0.90 (0.56-1.45) | 1.09 (0.70-1.69) | 0.69 (0.41-1.15) | 0.21 |
| Margarine | 1.00 | 0.91 (0.58-1.43) | 0.69 (0.44-1.10) | 0.84 (0.53-1.33) | 0.52 |
| Corn oil | 1.00 | 1.07 (0.60-1.90) | 1.26 (0.75-2.09) | 0.71 (0.38-1.32) | 0.40 |
| Canola oil | 1.00 | 1.52 (0.88-2.62) | 1.10 (0.67-1.83) | 1.09 (0.67-1.79) | 0.66 |
| Olive oil | 1.00 | 0.78 (0.43-1.40) | 0.78 (0.45-1.34) | 0.73 (0.42-1.27) | 0.20 |
| Diabetes |  |  |  |  |  |
| Butter | 1.00 | 0.98 (0.80-1.20) | 0.96 (0.80-1.16) | 1.20 (1.00-1.43) | 0.063 |
| Margarine | 1.00 | 1.04 (0.85-1.27) | 1.11 (0.93-1.34) | 1.19 (0.99-1.44) | 0.045 |
| Corn oil | 1.00 | 0.86 (0.66-1.12) | 1.02 (0.83-1.25) | 1.01 (0.84-1.23) | 0.87 |
| Canola oil | 1.00 | 0.97 (0.75-1.25) | 0.99 (0.81-1.22) | 0.89 (0.74-1.07) | 0.23 |
| Olive oil | 1.00 | 0.94 (0.73-1.22) | 0.94 (0.76-1.16) | 0.85 (0.70-1.04) | 0.11 |
| Infections |  |  |  |  |  |
| Butter | 1.00 | 0.89 (0.70-1.14) | 1.23 (1.00-1.51) | 1.17 (0.94-1.46) | 0.063 |
| Margarine | 1.00 | 1.03 (0.82-1.29) | 1.04 (0.84-1.29) | 1.02 (0.81-1.28) | 0.97 |
| Corn oil | 1.00 | 0.98 (0.72-1.32) | 1.32 (1.06-1.66) | 0.88 (0.68-1.12) | 0.60 |
| Canola oil | 1.00 | 0.72 (0.52-0.99) | 1.07 (0.84-1.36) | 0.86 (0.68-1.10) | 0.29 |
| Olive oil | 1.00 | 1.10 (0.82-1.47) | 0.88 (0.69-1.12) | 1.02 (0.81-1.29) | 0.97 |
| Kidney disease |  |  |  |  |  |
| Butter | 1.00 | 0.98 (0.70-1.37) | 1.09 (0.82-1.47) | 1.17 (0.87-1.55) | 0.26 |
| Margarine | 1.00 | 0.80 (0.58-1.11) | 1.07 (0.80-1.43) | 1.25 (0.93-1.67) | 0.012 |
| Corn oil | 1.00 | 0.91 (0.62-1.35) | 0.68 (0.46-1.02) | 1.04 (0.78-1.39) | 0.94 |
| Canola oil | 1.00 | 1.03 (0.70-1.52) | 0.86 (0.59-1.25) | 0.90 (0.67-1.22) | 0.43 |
| Olive oil | 1.00 | 0.95 (0.65-1.41) | 0.70 (0.47-1.05) | 1.02 (0.76-1.37) | 0.87 |
| Chronic liver disease |  |  |  |  |  |
| Butter | 1.00 | 0.91 (0.66-1.27) | 1.29 (0.99-1.67) | 1.41 (1.06-1.86) | 0.008 |
| Margarine | 1.00 | 0.87 (0.65-1.16) | 1.07 (0.81-1.40) | 0.80 (0.58-1.10) | 0.32 |
| Corn oil | 1.00 | 1.04 (0.70-1.54) | 1.15 (0.83-1.60) | 1.15 (0.84-1.57) | 0.32 |
| Canola oil | 1.00 | 0.56 (0.36-0.88) | 0.98 (0.69-1.40) | 0.97 (0.70-1.36) | 0.79 |
| Olive oil | 1.00 | 1.28 (0.88-1.86) | 0.92 (0.66-1.27) | 0.94 (0.69-1.28) | 0.63 |
| Other causes |  |  |  |  |  |
| Butter | 1.00 | 1.03 (0.96-1.11) | 1.08 (1.01-1.15) | 1.12 (1.04-1.20) | 0.002 |
| Margarine | 1.00 | 1.00 (0.93-1.07) | 1.00 (0.93-1.07) | 1.08 (1.00-1.16) | 0.011 |
| Corn oil | 1.00 | 0.91 (0.83-1.00) | 0.97 (0.89-1.06) | 0.98 (0.90-1.06) | 0.48 |
| Canola oil | 1.00 | 0.95 (0.86-1.04) | 0.93 (0.86-1.01) | 0.92 (0.85-0.99) | 0.010 |
| Olive oil | 1.00 | 0.97 (0.88-1.06) | 0.95 (0.87-1.03) | 0.99 (0.92-1.06) | 0.52 |

CI, confidence interval; HR, hazard ratio; T, tertile. HRs were adjusted for age, sex, BMI, race, education, marital status, household income, smoking, alcohol, vigorous physical activity, usual activity at work, perceived health condition, history of heart disease, stroke, diabetes, and cancer at baseline, Healthy Eating Index-2015, total energy intake, and consumption of remaining oils where appropriate (butter, margarine, lard, corn oil, canola oil, olive oil, and other vegetable oils).
